# VEGF pathway inhibition potentiates PARP inhibitor efficacy in ovarian cancer independent of BRCA status

**DOI:** 10.1186/s13045-021-01196-x

**Published:** 2021-11-06

**Authors:** Francesca Bizzaro, Ilaria Fuso Nerini, Molly A. Taylor, Alessia Anastasia, Massimo Russo, Giovanna Damia, Federica Guffanti, Francesca Guana, Paola Ostano, Lucia Minoli, Maureen M. Hattersley, Stephanie Arnold, Antonio Ramos-Montoya, Stuart C. Williamson, Alessandro Galbiati, Jelena Urosevic, Elisabetta Leo, Ugo Cavallaro, Carmen Ghilardi, Simon T. Barry, Maria Rosa Bani, Raffaella Giavazzi

**Affiliations:** 1 Laboratory of Cancer Metastasis Therapeutics, Department of Oncology, Istituto di Ricerche Farmacologiche Mario Negri IRCCS, Via Mario Negri 2, 20156 Milan, Italy; 2 Early Oncology, AstraZeneca, Cambridge, UK; 3 Laboratory of Molecular Pharmacology, Istituto di Ricerche Farmacologiche Mario Negri IRCCS, Milan, Italy; 4 Laboratory of Cancer Genomics, Fondazione Edo ed Elvo Tempia Valenta, Biella, Italy; 5 Department of Veterinary Medicine, University of Milan, Milan, Italy; 6 Early Oncology, AstraZeneca, Boston, USA; 7 Unit of Gynecological Oncology Research, Istituto Europeo di Oncologia IRCCS, Milan, Italy; 8 Present Address: Laboratory of Cancer Pharmacology, IRCCS Humanitas Research Hospital, Rozzano, Milan, Italy

**Keywords:** Ovarian cancer, Patient-derived xenograft, PARP inhibitor, VEGF pathway inhibitor, BRCA, Olaparib, Cediranib

## Abstract

Poly ADP-ribose polymerase inhibitors (PARPi) have transformed ovarian cancer (OC) treatment, primarily for tumours deficient in homologous recombination repair. Combining VEGF-signalling inhibitors with PARPi has enhanced clinical benefit in OC. To study drivers of efficacy when combining PARP inhibition and VEGF-signalling, a cohort of patient-derived ovarian cancer xenografts (OC-PDXs), representative of the molecular characteristics and drug sensitivity of patient tumours, were treated with the PARPi olaparib and the VEGFR inhibitor cediranib at clinically relevant doses. The combination showed broad anti-tumour activity, reducing growth of all OC-PDXs, regardless of the homologous recombination repair (HRR) mutational status, with greater additive combination benefit in tumours poorly sensitive to platinum and olaparib. In orthotopic models, the combined treatment reduced tumour dissemination in the peritoneal cavity and prolonged survival. Enhanced combination benefit was independent of tumour cell expression of receptor tyrosine kinases targeted by cediranib, and not associated with change in expression of genes associated with DNA repair machinery. However, the combination of cediranib with olaparib was effective in reducing tumour vasculature in all the OC-PDXs. Collectively our data suggest that olaparib and cediranib act through complementary mechanisms affecting tumour cells and tumour microenvironment, respectively. This detailed analysis of the combined effect of VEGF-signalling and PARP inhibitors in OC-PDXs suggest that despite broad activity, there is no dominant common mechanistic inter-dependency driving therapeutic benefit.

**Supplementary Information:**

The online version contains supplementary material available at 10.1186/s13045-021-01196-x.

To the Editor,

Olaparib is a first-in-class poly ADP-ribose polymerase inhibitor (PARPi) for treatment of recurrent platinum-responding ovarian cancer (OC) [1] with deleterious mutations in *BRCA* and other homologous recombination repair (HRR) pathway components which sensitize tumors to PARPi [2]. To increase the clinical therapeutic activity of PARPi, combination potential with vascular endothelial growth factor (VEGF) inhibition has been tested in multiple trials [2, 3]. This has resulted in olaparib plus bevacizumab being approved for maintenance treatment of OC in homologous recombination deficiency (HRD)-positive tumors [4]. Combining olaparib and cediranib, a small molecule receptor tyrosine kinase (RTK) inhibitor targeting VEGFR1, VEGFR2, VEGFR3 and mast/stem cell growth factor receptor c-KIT [5], provided a survival benefit in patients with relapsed platinum-sensitive OC, including *BRCA* wild-type tumours [6]. Our preclinical study assessed the benefit of combining VEGF signalling and PARP inhibition across a broad panel of patient-derived ovarian cancer xenografts (OC-PDXs). Cediranib potentiated the antitumor activity of olaparib regardless of the tumour’s sensitivity to platinum and olaparib and BRCA1/2 mutational status.

A cohort of subcutaneous OC-PDXs representing diverse OC genotypes and phenotypes (Additional file 1: Figs. S1–S3) [7] were treated with olaparib [1] and cediranib [5], mirroring clinical exposure [8]. Cediranib enhanced olaparib antitumor activity in all OC-PDXs, regardless of tumour histotype, HRR mutational status, expression of cediranib-targeted RTKs, sensitivity to platinum or olaparib monotherapy. As shown in Fig. 1A, the combination therapy showed broader activity versus olaparib monotherapy (81% vs 50%; *P* < 0.0001; odds ratio of 3.9) across the OC-PDX panel, with greater antitumor benefit in tumours refractory to platinum and olaparib. Tumour control seen at 4 weeks (Fig. 1A) was maintained at 8 weeks (Additional file 1: Fig. S4) with no increased toxicity. Notably, the combination achieved a durable growth stabilization of the platinum/olaparib poorly responsive MNHOC182 and MNHOC18 tumours, which bear heterozygous mutations in *BRCA2* (Fig. 1B and Additional file 1: Fig. S2). In *BRCA*-mutated and platinum/olaparib-sensitive OC-PDXs, the combination achieved rapid and prolonged tumour regression and this was evident also in tumours that no longer responded to olaparib at rechallenge (i.e. MNHOC513, MNHOC511, MNHOC508, Additional file 1: Fig. S6).

**Fig. 1:**
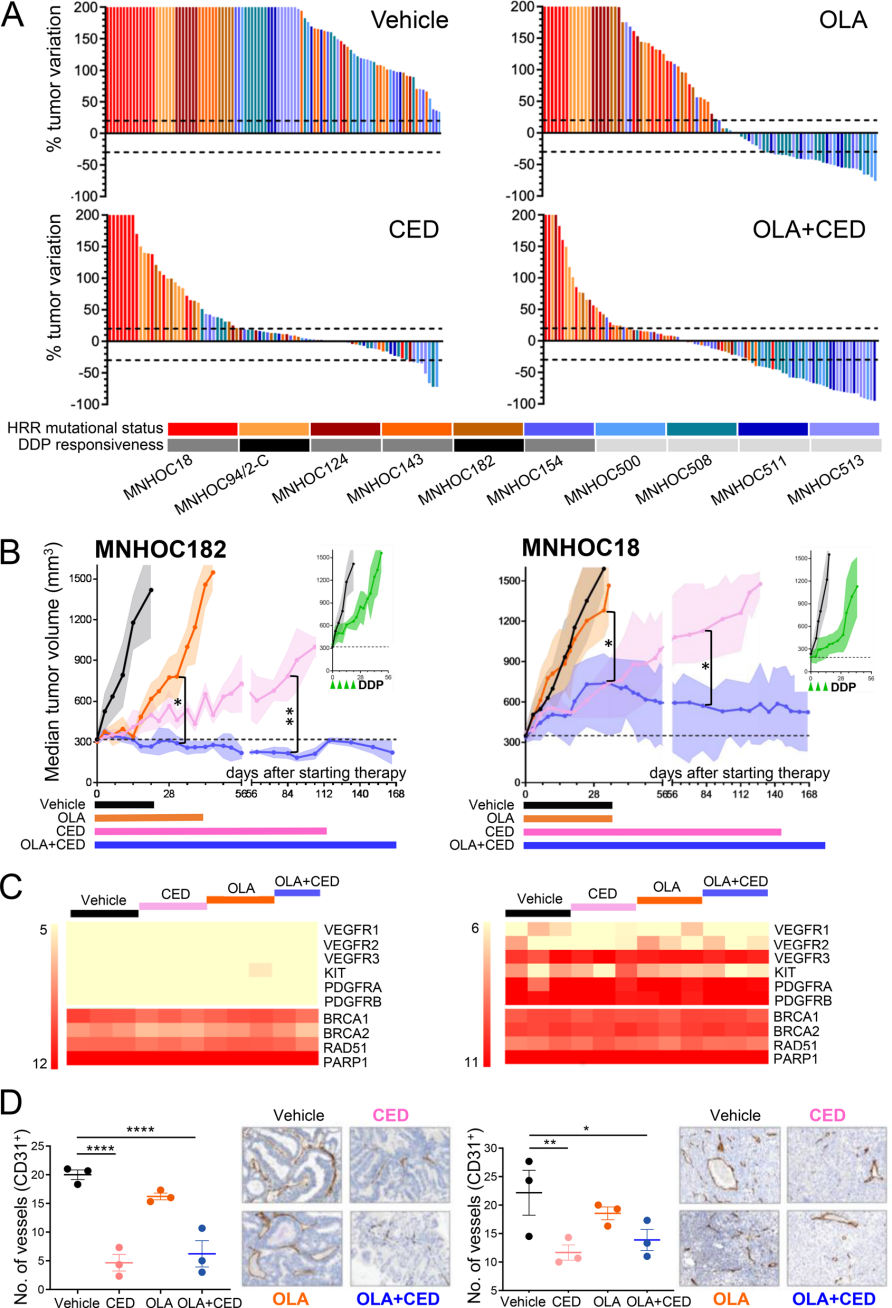
**Cediranib potentiates the antitumor activity of olaparib in OC-PDXs.** **A** OC-PDXs (*N* = 10) were transplanted subcutaneously in nude mice and treatment started when tumors reached approximately 300 mg. Olaparib (OLA 100 mg/kg) and cediranib (CED 3 mg/kg), as single agents or in combination (OLA + CED), were given orally by gavage once a day (QD) 5 days on and 2 days off (Q1 × 5). The change in tumour volume (compared with the tumour volume at treatment start, baseline for each mouse) after 4 weeks of treatment is shown in the waterfall plots (each vertical bar = one tumour): vehicle *N* = 100, CED *N* = 86, OLA *N* = 90, OLA + CED *N* = 98. RECIST category was determined/ as follow: change of tumour volume between + 25% and -30% was considered stable disease (SD), while below -30% was considered regressive disease (RD). The difference in objective response rate (ORR = the sum of RD and SD) of the combination therapy and olaparib monotherapy (81% vs 50%, respectively) was statistically significant (*P* < 0.0001; Wald test for logistic regression model) with an odds ratio of 3.9 (95% CI 2.05–7.41). Colours associated to each OC-PDX reflect the HRR mutational status (specified in Additional file 1: Fig. S1): tumours carrying a biallelic inactivating mutations in *BRCA1* or *BRCA2* genes (MNHOC154, MNHOC500, MNHOC508, MNHOC511, MNHOC513) are bluish; tumours being HRR wild-type (MNHOC124) or carrying heterozygous mutations in some HRR genes (MNHOC18, MNHOC94/2-C, MNHOC143, MNHOC182) are reddish. Sensitivity to cisplatin (DDP; cis-diaminedichloroplatinum) is also indicated: platinum-sensitive (T/C < 10%) light grey; marginally platinum-sensitive (T/C 10–50%) dark grey, platinum-resistant (T/C > 50%) black. **B-D** Treatment effects on MNHOC182 and MNHOC18 as exemplificative cases. **Treatment effects on tumour growth of MNHOC182 and MNHOC18 as exemplificative cases**. **B** Tumor growth, graphs are median tumour volume (mm^3^) ± median absolute deviation (MAD, shaded area). Coloured bars at the bottom indicate the study dosing period. DDP response (tested in the same experiment) is reported in the insert at the side. MNHOC182: Vehicle *N* = 5, CED *N* = 4, OLA *N* = 4, OLA + CED *N* = 5; MNHOC18: Vehicle *N* = 6, CED *N* = 6, OLA *N* = 6, OLA + CED *N* = 6. Differences in tumour volume were analysed by ANOVA and Tukey’s post-test (or t test when only two groups were compared) the days of measurement. *P < 0.05; **P < 0.01. **C** Heatmap of mRNA expression in MNHOC182 (left panel) and MNHOC18 (right panel) treated for 4 weeks. Log2 normalized values of 3 independent tumors/mice are shown. **D** Quantitative analyses and representative images (magnification 200x) of immunohistochemistry (IHC) staining for microvessel density (number of CD31^+^ vessels per mm^3^) after 4 weeks of treatment. MNHOC182 (left panel) and MNHOC18 (right panel). Statistic by ANOVA and Tukey’s post-test. *P < 0.05; **P < 0.01; ****P < 0.001. (Detailed methods in Additional file 2)

Extensive analysis was performed to identify potential drivers of combination efficacy. Consistent with its mode of action [9], cediranib dosed alone and in combination with olaparib significantly reduced OC-PDX tumour-associated vasculature (Fig. 1D and Additional file 1: Figs. S5, S7). Previous studies have suggested that cediranib-induced hypoxia sensitizes tumour cells to PARPi by reducing HRR gene expression [10], and that cediranib inhibits *BRCA* expression through platelet-derived growth factor receptor (PDGFR) inhibition, thus inducing a contextual tumour BRCAness [11]. Our analysis suggests that downregulation of HRR genes or modulation of PDGFRβ signalling is not a dominant effect underpinning the combination benefit. Specifically, under severe hypoxic conditions in vitro, we did not observe any consistent change in *RAD51* expression across a panel of OC cell lines (Additional file 1: Fig. S8). Moreover, downregulation of *BRCA* or *RAD51 *in vitro was only achieved at ≥ 3 μM concentrations of cediranib (Additional file 1: Fig. S9A,B), far higher than the clinical exposure. In contrast to other studies *BRCA* or *RAD51* modulation was not prevented by silencing of *E2F4* (Additional file 1: Fig. S9B), the transcription factors suggested to regulate expression via PDGFRβ [11]. Consistent with this, HRR gene expression was not modulated in the OV2022 PDX by either short or long term dosing, despite increased combination benefit (Additional file 1: Fig. S10). Likewise in OC-PDXs, no common changes in the expression of a broad panel of genes (including HRR ones) by cediranib or the combination were found (Fig. 1C and Additional file 1: Figs. S5, S11), nor there was any association between RTK basal expression (Additional file 1: Fig. S3A) and sensitivity to treatments at the clinically relevant dose of cediranib (Figs. 1, 2). Indeed, cediranib addition to olaparib significantly impaired the growth of MNHOC182 tumors (Fig. 1B) which have low/null *PDGFRB* expression (Fig. 1C). Pharmacokinetic modeling of the cediranib exposure profile in patients showed that robust inhibition of PDGFR is not achieved at clinically relevant doses [12] and consistent with our findings, olaparib efficacy is also enhanced by bevacizumab [2], which does not inhibit PDGFR, further supporting the conclusion that targeting PDGFR signalling is not a driver of combination benefit.

**Fig. 2:**
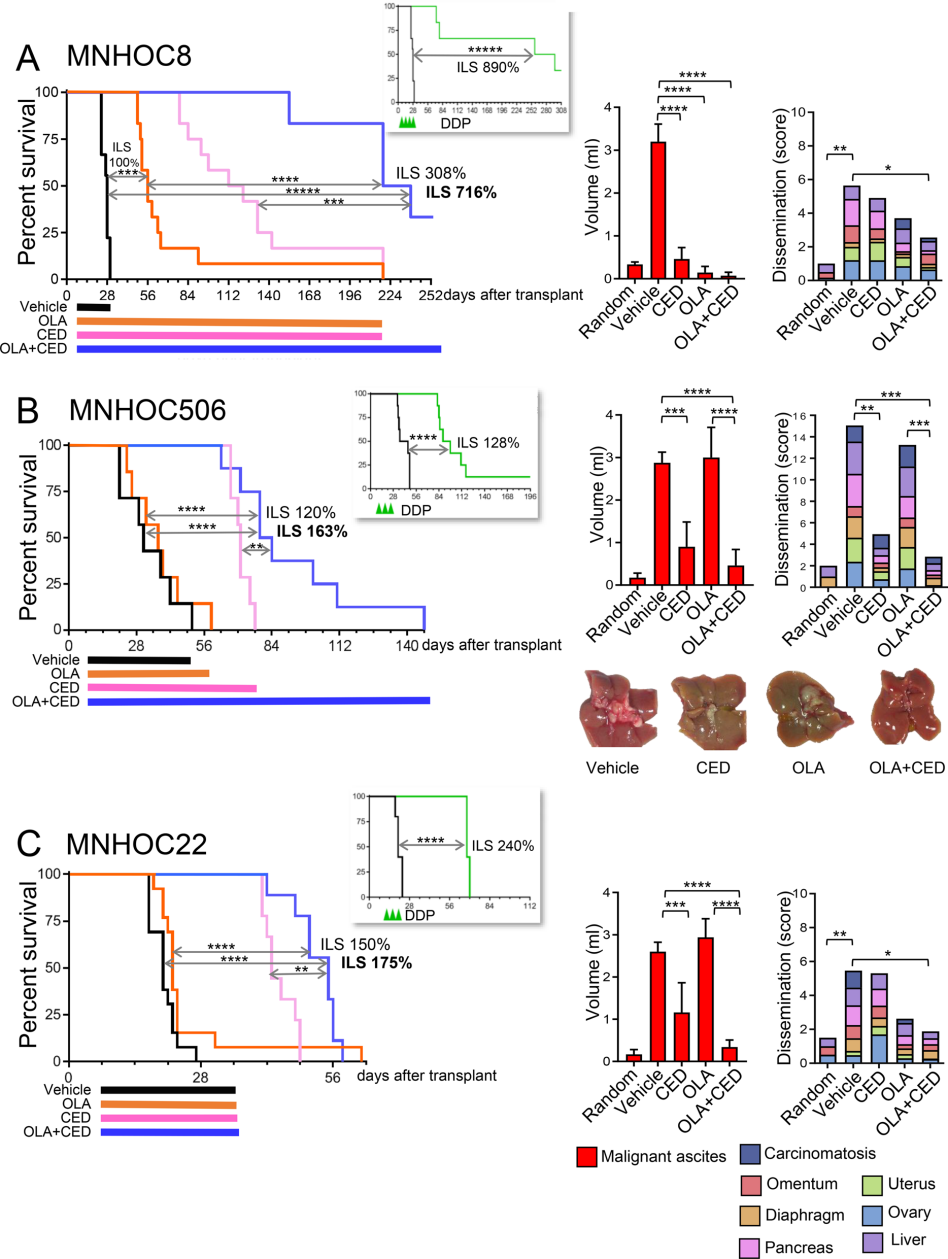
**Cediranib combined with olaparib reduces tumor dissemination and prologs survival in orthotopic OC-PDXs**. Drug effect in orthotopic OC-PDXs. Olaparib (100 mg/kg) and cediranib (3 mg/kg), monotherapy or combined, were given orally by gavage QD (Q1 × 5) until progression. **Left panels**: Survival (Kaplan Meier) curves of tumour bearing mice; the benefit calculated as increment of lifespan (ILS%) of disease bearing mice is indicated. Coloured bars at the bottom indicate the study dosing period. DDP response is reported in insert at the side. **Right panels**: Abdominal tumour burden (i.e. volume of ascites in the peritoneal cavity and organ dissemination) assessed after 4 weeks of treatment. Ascites and dissemination data are mean ± SD. “Random” indicates tumour burden at randomization (start of treatment). Statistic by Wilcoxon rank-sum test/log-rank test (left panels) or ANOVA and Tukey’s post-test (right panels). *P < 0.05; **P < 0.01; ***P < 0.005; ****P < 0.001; *****P < 0.0001. **A** **MNHOC8** (wild-type for *BRCA* genes Additional file 1: Fig. S1B but lacking *BRCA1* mRNA due to promoter methylation Additional file 1: Fig. S3): mice were randomized 7 days after intraperitoneal tumor transplant. Vehicle *N* = 9, CED *N* = 12, OLA *N* = 12, OLA + CED *N* = 6. **B** **MNHOC506** (wild-type for HRR genes; Additional file 1: Fig. S1B): mice were randomized 9 days after intraperitoneal tumor transplant. Vehicle *N* = 7, CED *N* = 7, OLA *N* = 7, OLA + CED *N* = 8. Representative images of tumor dissemination in liver are reported. **C** **MNHOC22** (carrying a homozygous pathogenic nonsense mutation in *BRCA1*, truncating the protein; Additional file 1: Fig. S1): mice were randomized 6 days after intraperitoneal tumor transplant. Vehicle *N* = 13, CED *N* = 9, OLA *N* = 13, OLA + CED *N* = 9. (Detailed methods in Additional file 2)

The benefit of the combination in controlling tumour progression was confirmed in three disease relevant orthotopic OC-PDXs (Fig. 2) that mimic human disease with dissemination occurring in the mouse peritoneal cavity [7]. Cediranib addition prolonged the lifespan of mice bearing the platinum/olaparib-responsive MNHOC8 (Fig. 2A) lacking BRCA1 expression (Additional file 1: Fig. S3) and most importantly the olaparib-resistant MNHOC506 and MNHOC22 (Fig. 2B, C). Analysis at 4 week of treatment, highlighted the need of both drugs controlling ascites formation and tumour dissemination (Fig. 2, right panels), resulting in survival increase. Tumour regrowth at therapy discontinuation (Additional file 1: Fig. S12) suggests that sustained treatment is required to maximise prolonged tumour control. Collectively these data suggest that PARPi and VEGFi target complementary mechanisms to reduce tumour and that continuous therapy may be clinically important to restrain tumour progression.

In summary, we show that the addition of cediranib enhances the antitumor efficacy of olaparib in both olaparib-sensitive and olaparib-resistant OCs. Pre-clinical activity was not associated with a mechanism priming response to cediranib or olaparib. Rather the agents appear to be complementary, with olaparib impacting DNA repair and cediranib modulating tumour vasculature, to increase response across the entire OC-PDXs panel. Our extensive experimental in vivo efficacy data reproduce clinical observations, while our comprehensive mechanistic analysis suggests the combination effect is largely driven by targeting independent mechanisms, rather than inducing specific changes that prime response to a partner drug.

## Supplementary Information

**Additional file 1.** **Supplementary Figure S1.** Characteristics of the OC-PDXs used for drug efficacy testing. **Supplementary Figure S2.** Haploinsufficiency of the BRCA2 mutation in MNHOC182 and MNHOC18. **Supplementary Figure S3.** Transcriptional status of key genes in OC-PDXs and BRCA1 promoter methylation. **Supplementary Figure S4.** Antitumor activity of the olaparib and cediranib combination therapy at 8 weeks of treatment. **Supplementary Figure S5.** Cediranib reduced MNOC124 tumour associated vasculature but did not affect the expression of HRR genes. **Supplementary Figure S6.** Rapid and prolonged tumour response by the combination olaparib and cediranib in platinum-sensitive and olaparib-sensitive OC-PDXs. **Supplementary Figure S7.** Reduction of tumour associated vasculature by cediranib in the OC-PDXs cohort used for drug efficacy testing. **Supplementary Figure S8.** Hypoxia does not trigger the downregulation of HRR in ovarian cancer cell lines. **Supplementary Figure S9.** RAD51 downregulation is not related to PDGFRB pathway. **Supplementary Figure S10.** The combination olaparib and cediranib demonstrated greater efficacy than either monotherapy in OV2022 tumours: no therapy-induced changes in HRR genes could be detected. **Supplementary Figure S11.** No common changes in gene expression by cediranib treatment were identified in OC-PDXs that benefit from the combination therapy. **Supplementary Figure S12.** Survival advantage is lost upon treatment interruption. **Supplementary Table S1.** List of genes analysed by Fluidigm high-throughput gene expression analysis.

**Additional file 2.** Methods.

## Data Availability

Data supporting the findings of this study are available within the article and Additional Files. The datasets generated during the current study are available from the corresponding author on reasonable request and with permission of AstraZeneca.

## Abbreviations

CED: Cediranib
cKIT: KIT proto-oncogene, mast/stem cell growth factor receptor
DDP: Cis-diaminedichloroplatinum, cisplatin
HRD: Homologous recombination deficiency
HRR: Homologous recombination repair
IHC: Immunohistochemistry
ILS: Increment of lifespan
OC: Ovarian cancer
OC-PDX: Patient-derived ovarian cancer xenograft
OLA: Olaparib
PARP: Poly ADP-ribose polymerase
PARPi: Poly ADP-ribose polymerase inhibitor
PDGFR: Platelet-derived growth factor receptor
PK: Pharmacokinetics
QD: *quaque die*, Once a day
RTK: Receptor tyrosine kinase
VEGF: Vascular endothelial growth factor
VEGFR: Vascular endothelial growth factor receptor
