# Supplementary material for: VEGF pathway inhibition potentiates PARP inhibitor efficacy in ovarian cancer independent of BRCA status
Source: J Hematol Oncol. 2021 Nov 6;14:186. doi: 10.1186/s13045-021-01196-x (PMC8572452; doi:10.1186/s13045-021-01196-x)
Supplement: Supplementary file 1 — Additional file 1. Supplementary Figure S1. Characteristics of the OC-PDXs used for drug efficacy testing. Supplementary Figure S2. Haploinsufficiency of the BRCA2 mutation in MNHOC182 and MNHOC18. Supplementary Figure S3. Transcriptional status of key genes in OC-PDXs and BRCA1 promoter methylation. Supplementary Figure S4. Antitumor activity of the olaparib and cediranib combination therapy at 8 weeks of treatment. Supplementary Figure S5. Cediranib reduced MNOC124 tumour associated vasculature but did not affect the expression of HRR genes. Supplementary Figure S6. Rapid and prolonged tumour response by the combination olaparib and cediranib in platinum-sensitive and olaparib-sensitive OC-PDXs. Supplementary Figure S7. Reduction of tumour associated vasculature by cediranib in the OC-PDXs cohort used for drug efficacy testing. Supplementary Figure S8. Hypoxia does not trigger the downregulation of HRR in ovarian cancer cell lines. Supplementary Figure S9. RAD51 downregulation is not related to PDGFRB pathway. Supplementary Figure S10. The combination olaparib and cediranib demonstrated greater efficacy than either monotherapy in OV2022 tumours: no therapy-induced changes in HRR genes could be detected. Supplementary Figure S11. No common changes in gene expression by cediranib treatment were identified in OC-PDXs that benefit from the combination therapy. Supplementary Figure S12. Survival advantage is lost upon treatment interruption. Supplementary Table S1. List of genes analysed by Fluidigm high-throughput gene expression analysis. [file 13045_2021_1196_MOESM1_ESM.pdf]

ADDITIONAL FILE 1.

VEGF pathway inhibition potentiates PARP inhibitor efficacy in ovarian cancer independent of BRCA status.

Francesca Bizzaro, Ilaria Fuso Nerini, Molly A. Taylor, Alessia Anastasia, Massimo Russo, Giovanna Damia, Federica Guffanti, Francesca Guana, Paola Ostano, Lucia Minoli, Maureen M. Hattersley, Stephanie Arnold, Antonio Ramos-Montoya, Stuart C. Williamson, Alessandro Galbiati, Jelena Urosevic, Elisabetta Leo, Ugo Cavallaro, Carmen Ghilardi, Simon T. Barry, Maria Rosa Bani, Raffaella Giavazzi

Additional data :

Supplementary Figure S1
Supplementary Figure S2
Supplementary Figure S3
Supplementary Figure S4
Supplementary Figure S5
Supplementary Figure S6
Supplementary Figure S7
Supplementary Figure S8
Supplementary Figure S9
Supplementary Figure S10
Supplementary Figure S11
Supplementary Figure S12
Supplementary Table 1

A

Subcutis OC-PDX	Histotype	OC-PDX somatic mutations				DDP responsiveness
		TP53	Other	BRCA1	BRCA2	
MNHOC18	HGS	p.C176F c.527G>T		wt	p.T1067A c.3199A>G NO site specific LOH	PPS
MNHOC94/2-C	CC	wt	PTEN p.R130* c.388C>T	wt	wt	R
MNHOC124	HGS	p.R175H c.524G>A		wt	wt	PPS
MNHOC143	HGS	p.V157D c.470T>A		wt	wt	PPS
MNHOC154	HGE	p.R282W c.844C>T		p.C61G c.181T>G	wt	PPS
MNHOC182	MUC	wt	KRAS p.G12D c.35 G>A	wt	p.M2393fs c.7177dupA No site specific LOH	R
MNHOC500	HGS	p.H214R c.641A>G		p.N363fs c.1088delA	wt	S
MNHOC508	HGS	c.782+1G>A (splice)		wt	p. L1908fs c.5722-5723delCT	S
MNHOC511	HGS	p.R175G c.523C>G		p.C64R c.190T>C	p.S2071T c.6212G>C	S
MNHOC513	HGS	p.F113fs c.338 delT		exon 23 loss/deletion Chr17:41197695	wt	S
**Ortothopic OC-PDX**						
MNHOC8	HGS	p.V172F c.514G>T		NO mRNA expression	wt	S
MNHOC22	HGS	c.993+1G>A (splice)		p.Q563* c.1687C>T	wt	S
MNHOC506	HGS	G266* c.796G>T		wt	wt	PPS

B

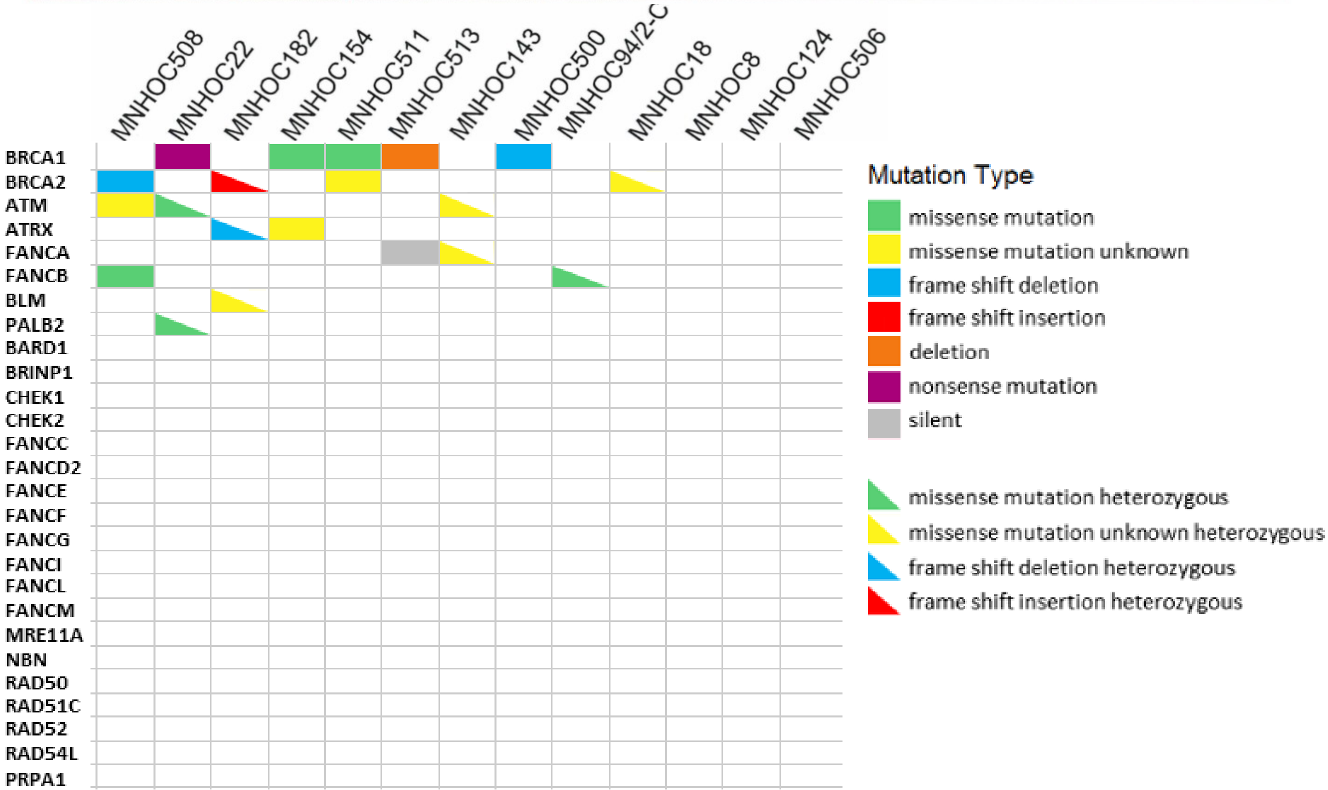

Supplementary Figure S1. OC-PDXs used for drug efficacy testing

From a panel of well-characterized OC-PDXs [7], 13 were selected to evaluate the efficacy of olaparib and cediranib combination therapy. For the study, 10 OC-PDXs were implanted subcutaneously and 3 orthotopically in the peritoneal cavity of nude mice to recapitulate the dissemination pattern of ovarian cancer in patients.

A OC-PDXs characteristics: histotype, TP53 and other OC genes (e.g. KRAS, PTEN), BRCAI and BRCA2 mutations, sensitivity to DDP (S= sensitive, i.e. T/C<10% or ILS> 150%; PPS= partially platinum -sensitive, i.e. T/C 10-50% or ILS 40-150%, R= resistant, i.e. T/C>50% or ILS<40%).

B Heat map of OC-PDXs somatic mutations in the HRR genes (described in Coleman et al. Lancet 2017) assessed by whole exome sequencing. Methods detailed in Additional File 2.

OC-PDXs carrying a biallelic inactivating mutations in BRCA1 or BRCA2 (MNHOC154, MNHOC500, MNHOC508, MNHOC511, MNHOC513, MNHOC22) were considered HRR-deficient (bluish); tumours either HRR wild type (MNHOC124, MNHOC506) or carrying heterozygous mutations in some HRR genes (MNHOC18, MNHOC94/2-C, MNHOC143, MNHOC182) were considered HRR-proficient (reddish). Despite carrying no alteration in any HRR genes, MNHOC8 was considered HRR-deficient due to lack of BRCA1 expression (Supplementary Fig. S3A) and methylation of the promoter (Supplementary Fig. S3B).

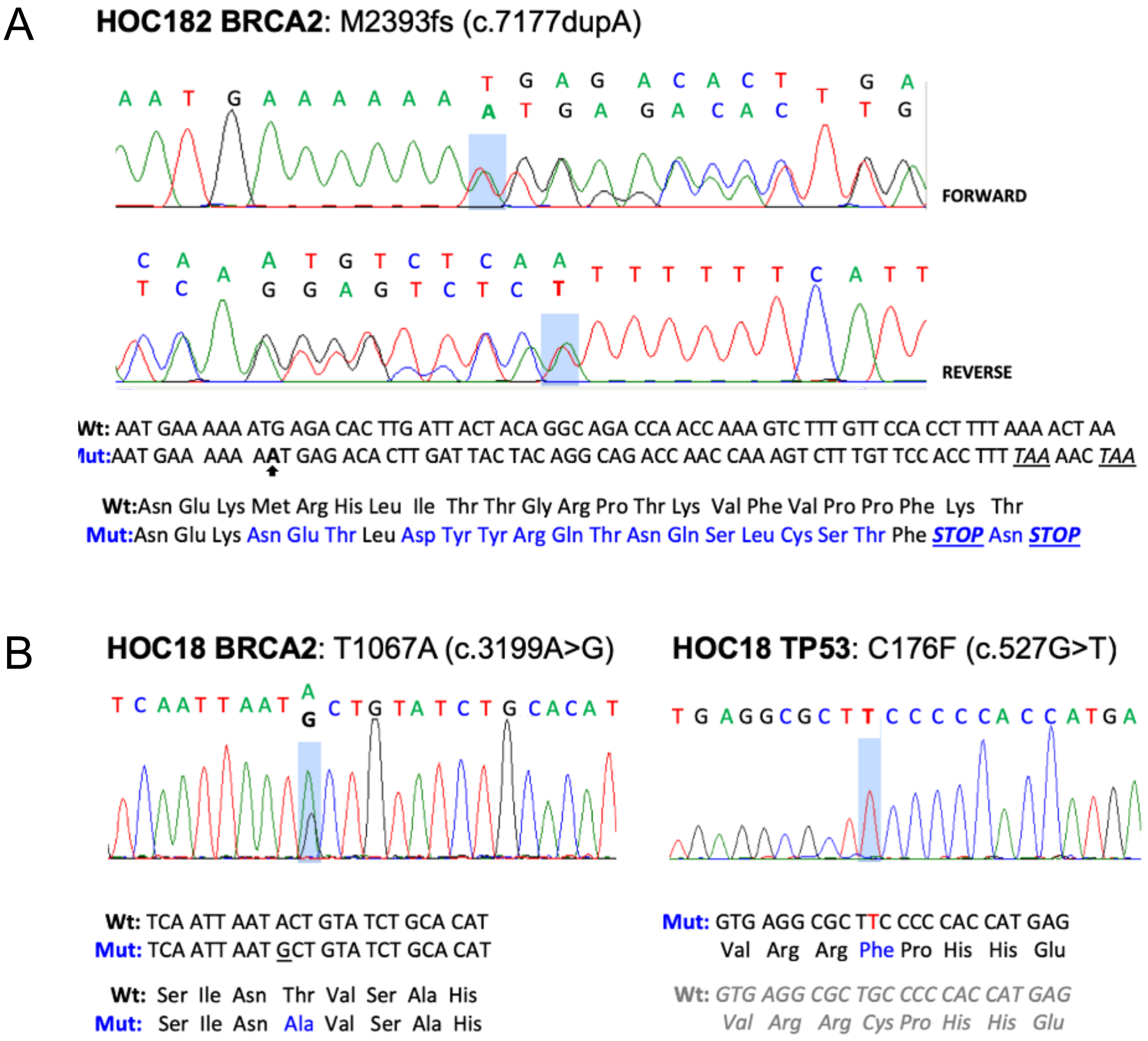

Supplementary Figure S2. Haploinsufficiency of the BRCA2 mutation in MNHOC182 and MNHOC18 OC-PDXs

A MNHOC182 and **B** MNHOC18 were genotyped for BRCA2 mutation and site-specific loss of heterozygosis (LOH). Methods detailed in Additional File 2. Sanger sequencing electropherograms are shown.

Sequencing of the mRNA confirmed that the BRCA2 mutated locus are in heterozygous combination enabling functional protein (encoded by the wild type allele) to be produced.

A

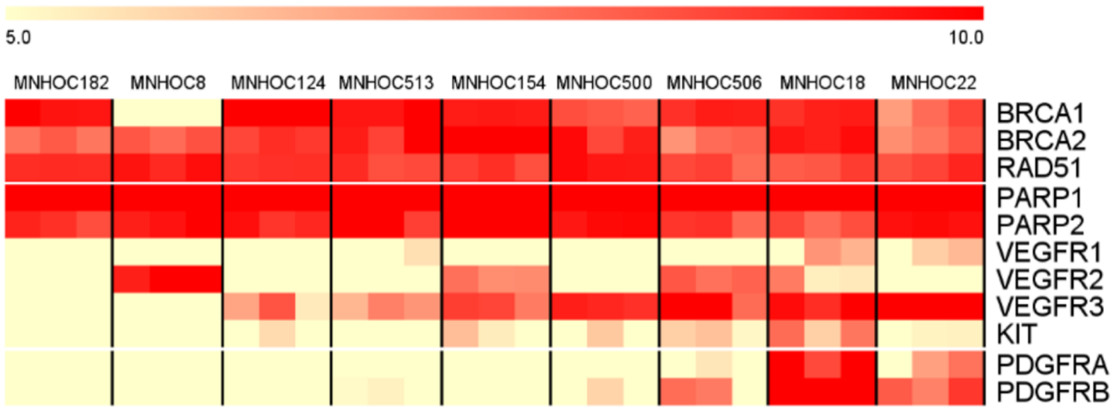

B

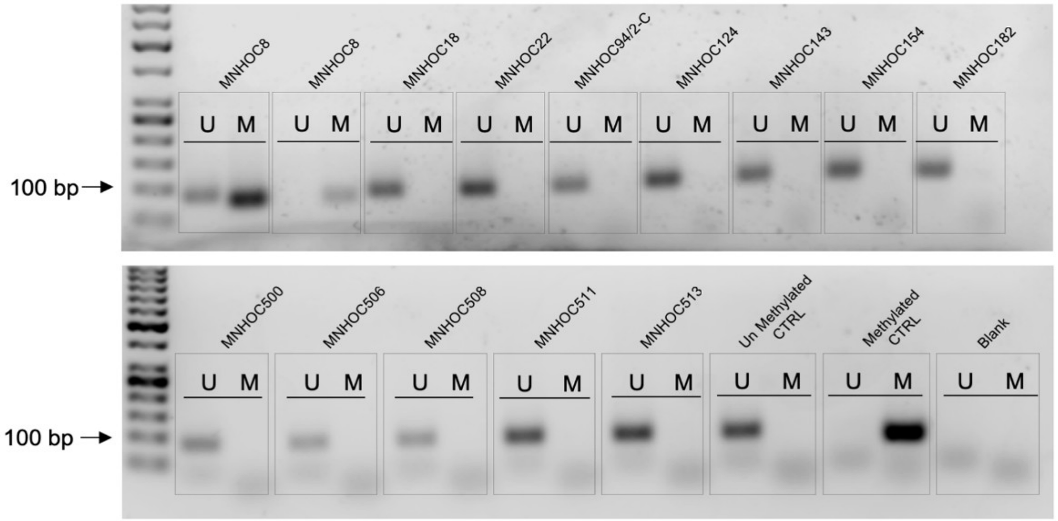

Supplementary Figure S3. Transcriptional status of key genes in OC-PDXs

A Heat map of mRNA expression by the OC-PDXs: HRR key genes (BRCA1, BRCA2, RAD51), olaparib targets (PARP1 and PARP2) and cediranib targets (VEGFR1, VEGFR2, VEGFR3, cKIT, PDGFRA, PDGFRB). Shown is the log2 normalized values of three independent tumours/mice.

B BRCA1 promoter methylation assessed by methylation-specific PCR. Unmethylated (U) amplicon size: 86 bp; methylated (M) amplicon size: 75 bp. Methods detailed in Additional File 2.

The expression of RTKs targeted by cediranib was highly variable across the OC-PDXs. *VEGFR3* is much lower in MNHOC182 and MNHOC8 (log2 being 2.3 and 3.8 respectively) compared to the other OC-PDXs (log2 from 5.4 to 11.9); *PDGFRA* and *PDGFRB* were much higher in MNHOC18 (log2 being 10.3 and 11.2 respectively) compared to the other OC-PDXs (log2 from 0 to 7.8 and from 0 to 8.9 respectively).
No major difference in basal expression of *BRCA2* and *RAD51* or *PARP1* and *PARP2* was observed across the OC-PDXs. At difference, *BRCA1* expression was suppressed in MNHOC8 (log2 being 1.14 compared to the other OC-PDXs log2 from 6.9 to 11.6) which is the only OC-PDX with BRCA1 promoter hypermethylated.

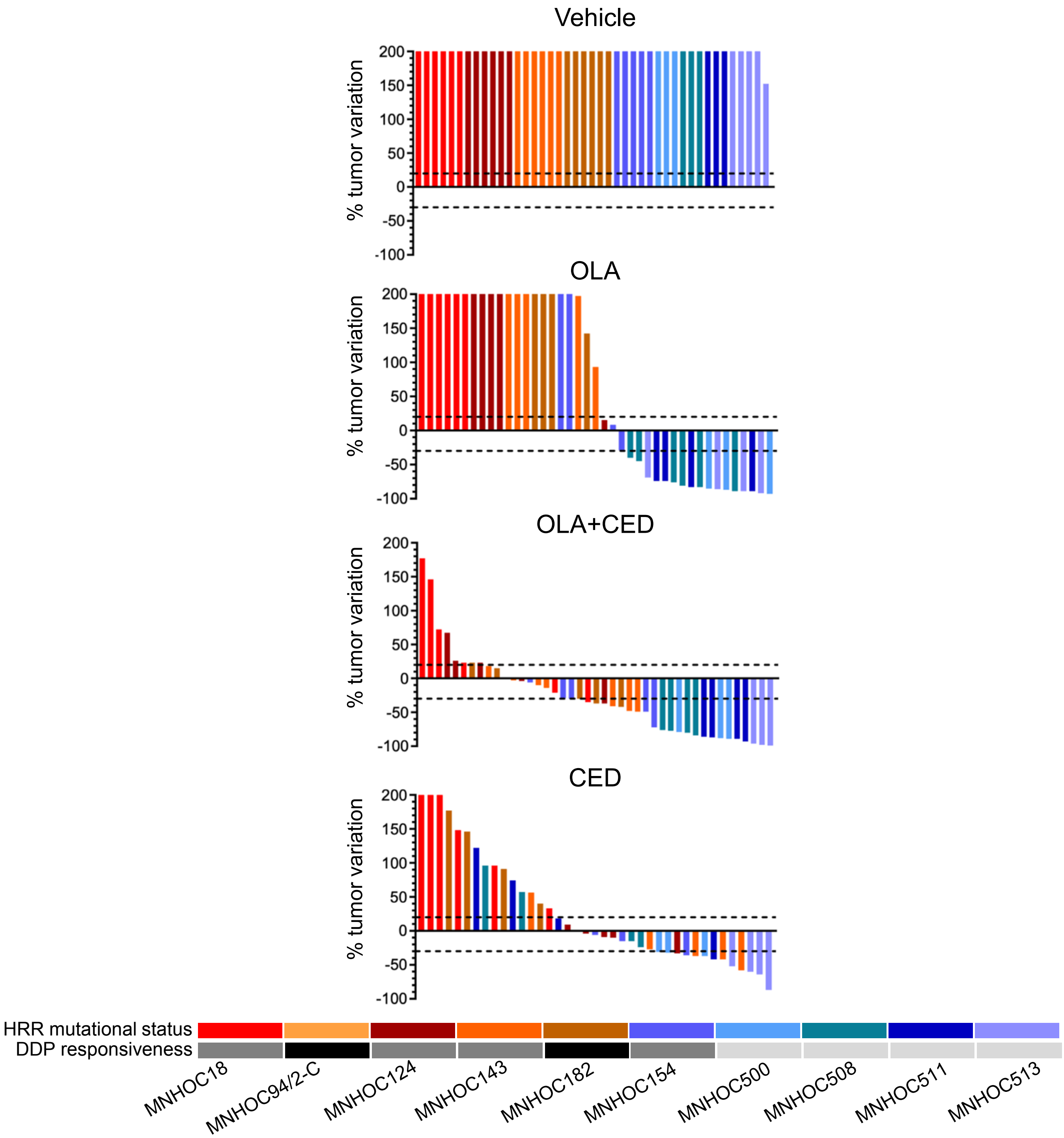

Supplementary Figure S4. Antitumor activity of the combination olaparib and cediranib at 8 weeks of treatment

OC-PDXs transplanted subcutaneously in nude were treated as in Fig.1. Shown in the waterfall plots are the tumour volume changes (compared with the tumour volume at treatment start) for each mouse after 8 weeks of treatment (each vertical bar = one tumour). Experimental groups: vehicle; olaparib (OLA); combination (OLA+CED); cediranib (CED). Methods detailed in Additional File 2. A change of tumour volume between 25% and -30% indicate stable disease, while below -30% indicate regressive disease. A change bigger than 25% denotes progressive disease.

The antitumor activity of the combination seen at 4 weeks (Fig. 1A) was maintained following long-term treatment (8 weeks) impairing the growth of tumours not responsive to olaparib and poorly responsive to cisplatin (reddish).

A

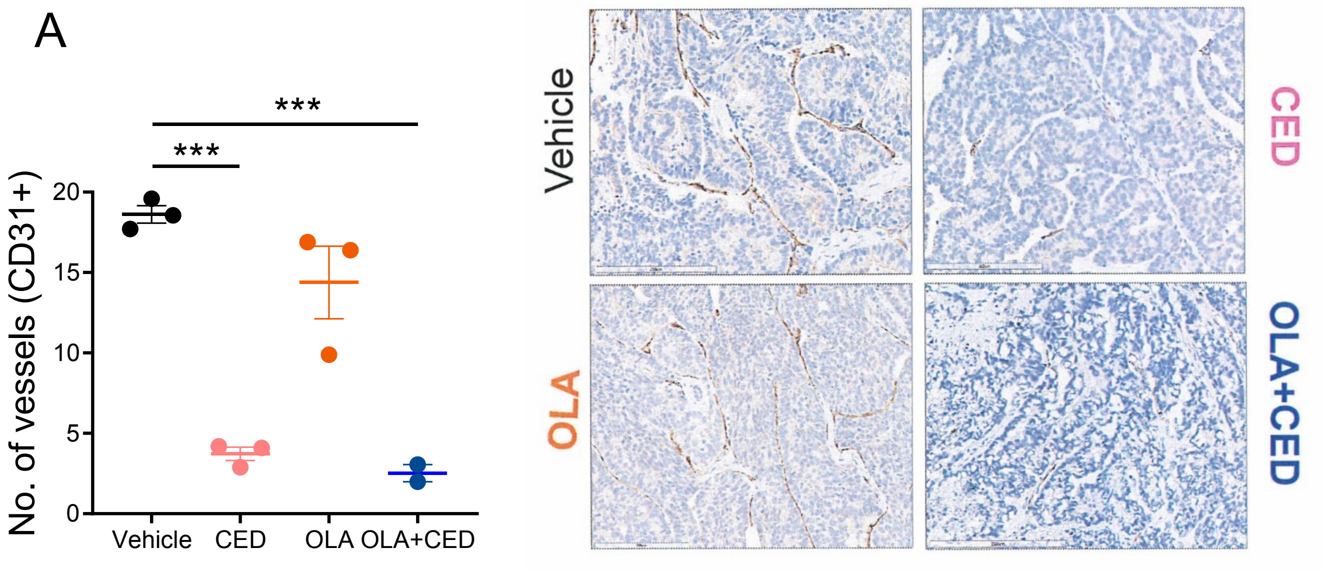

B

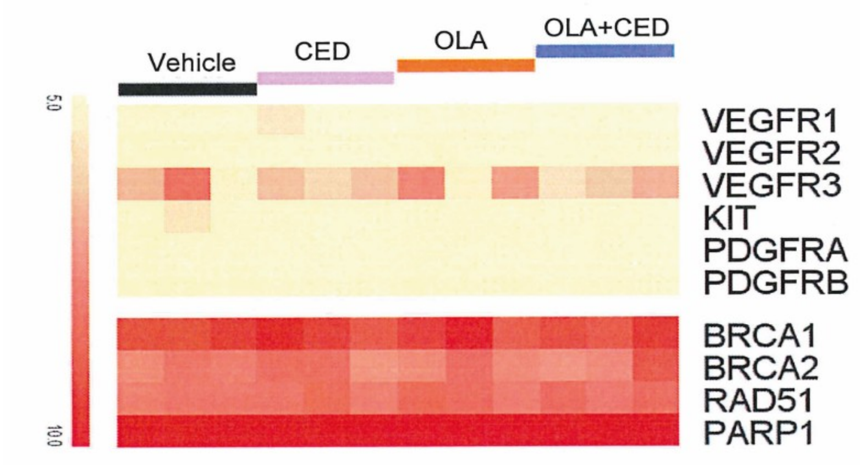

Supplementary Figure S5. Cediranib reduced tumour associated vasculature but did not affect HRR gene expression by MNHOC124

Subcutaneous growing HOC124 tumours were harvested after 4 weeks of treatment with olaparib, cediranib or the combination.

A Quantitative analyses and representative images (magnification 200x) of IHC staining for microvessel density (number of CD3 l^{+} vessels per mm^{3}). Statistic by ANOVA and Tuckey's post-test. ***p<0.005.

B Heat map of mRNA expression. Log2 normalized values of 3 independent tumours/mice are shown.

Tumour-associated vasculature was significantly reduced by cediranib, single agent or in combination, however, *BRCA1*, *BRCA2* and *RAD51* transcripts were not affected. Likewise, the expression of RTK targeted by cediranib (VEGFR1, VEGFR2, VEGFR3, KIT) were not modulated by any treatment, nor were *PDGFRA* and *PDGFRB*, or the olaparib target *PARP1.*

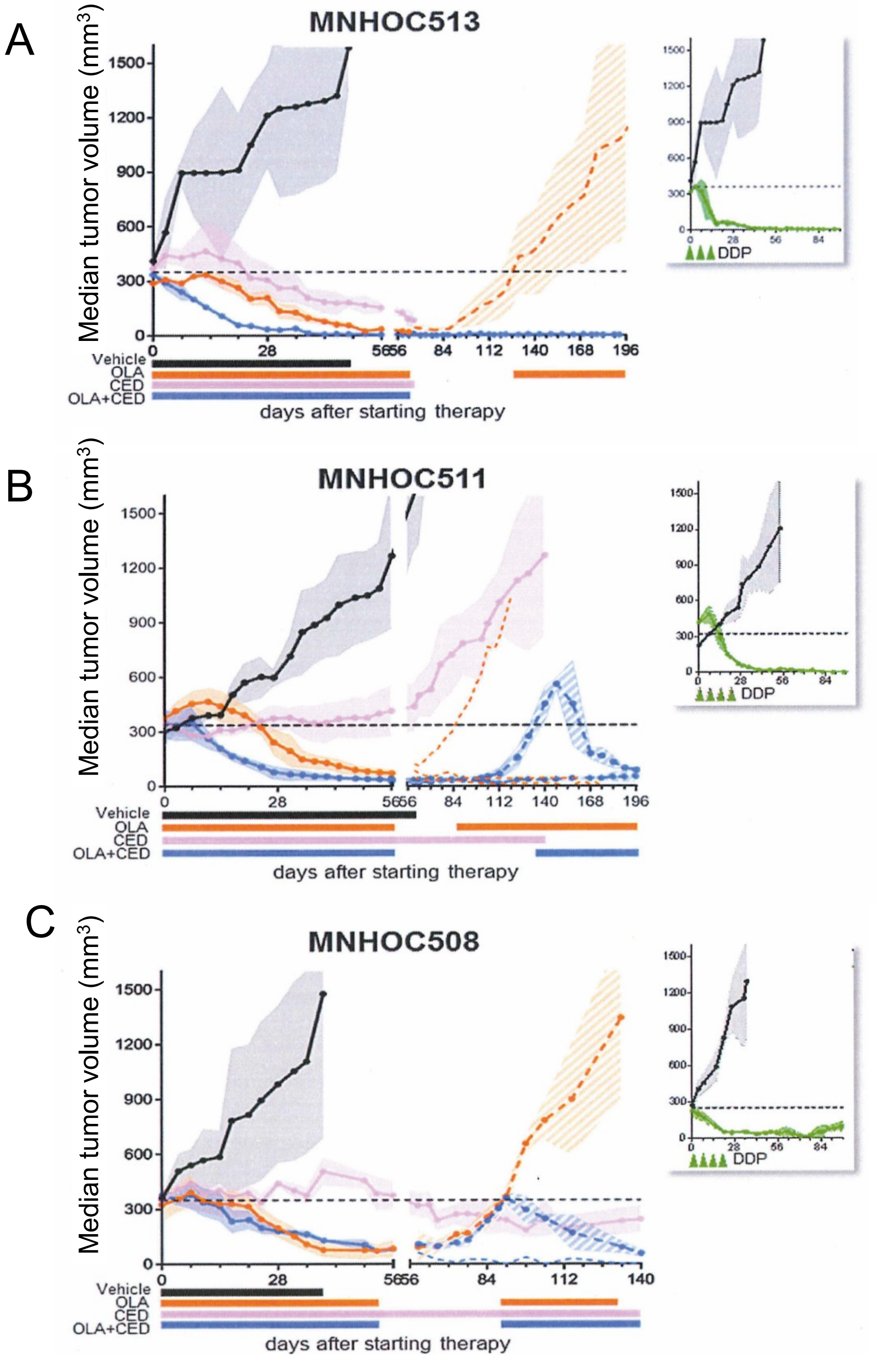

Supplementary Figure S6. Rapid and prolonged tumour responses by olaparib and cediranib combination in platinum-sensitive and olaparib-sensitive OC-PDXs

Effect of treatments on MNHOC513 (**A**), MNHOC511 (**B**) and MNHOC508 (**C**), as exemplificative cases of olaparib/DDP-sensitive OC-PDX. Treatments as in Fig. 1; methods detailed in Additional File 2. Graphs are median tumour volume (mm^3) ± median absolute deviation (MAD, shaded area). Dotted lines represent the re-growing tumours. Coloured bars at the bottom indicate the study-dosing period. The DDP response is shown in the insert at the side.

BRCA-mutated platinum/olaparib-sensitive OC-PDXs experienced a rapid tumour regression and the benefit of the combination was evident in tumours re-grown upon treatment withdrawal. Following cessation of treatment (at regression), all the MNHOC513 bearing mice dosed with the combination remained tumour free while olaparib-treated tumours immediately re-grew and did not respond to olaparib re-challenge (A). MNHOC511 tumours treated with the combination regressed much faster and the re-grown tumours were sensitive to the re-challenge while no further response was obtained with olaparib. Likewise, MNHOC508 tumours that re-grew following cessation of treatment (at regression) were still sensitive to the combination while olaparib treated tumours became resistant.

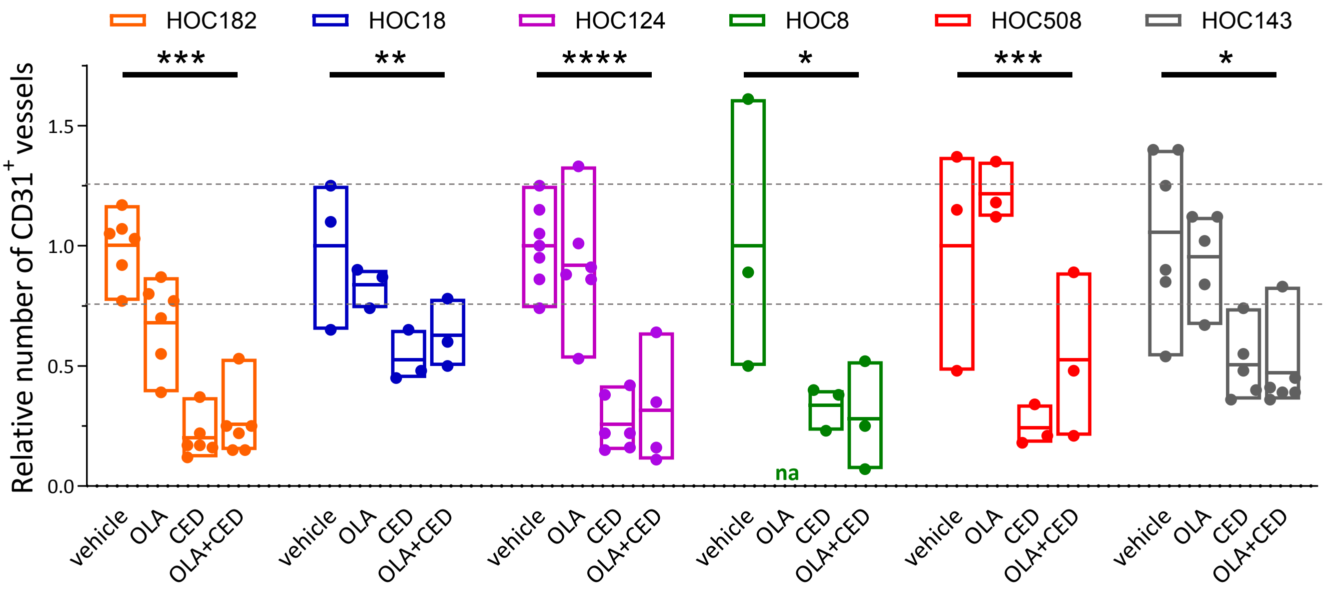

Supplementary Figure S7. Reduction of tumour-associated vasculature by cediranib.
After 4 weeks of treatment, tumours were removed, immunostained and microvessel density assessed as described in Additional File 2. The number of $CD31^+$ vessels per mm^3 (MDV) varied greatly across the OC-PDXs. For every OC-PDX analysed the MDV (mean value) of the vehicle treated tumours was arbitrarily assumed as reference and for each treated tumours the "relative number of $CD31^+$ vessels per mm^3" was calculated and is shown in the graph (where each dot represents a tumour). Reduction ranged from 35% to 70% (HOC18 and HOC182 respectively). Statistics by ANOVA and Tuckey's multiple comparisons test. *p<0.05; **p<0.01; ***p<0.005, ****p<0.001.

Cediranib contributes to the overall antitumor efficacy of the combination by significantly reducing tumour-associated vasculature across all the OC-PDXs when dosed together with olaparib.

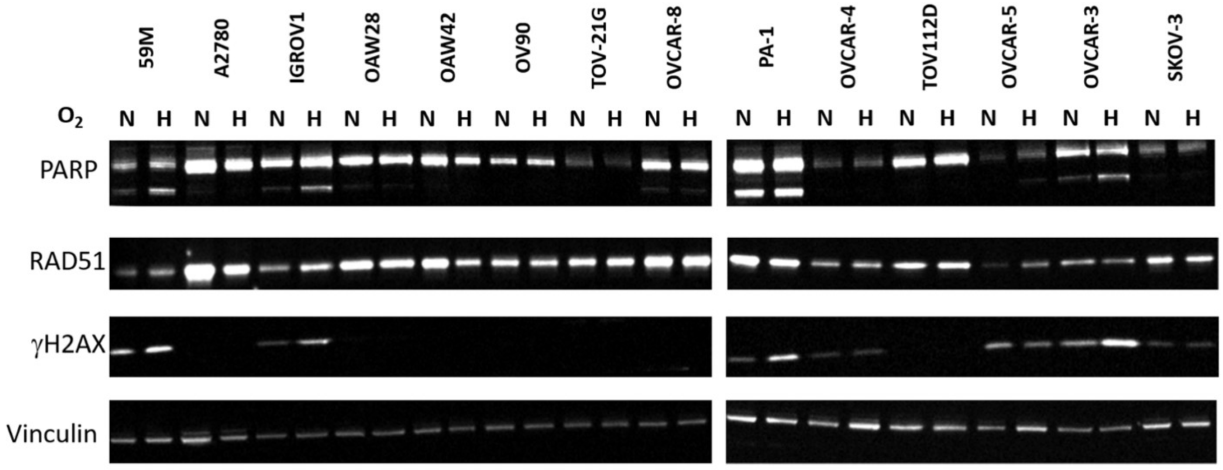

N-Normoxic (20% O_2); H-Hypoxic (0.1% O_2) for 24 hours

Supplementary Figure S8. Hypoxia does not trigger the down regulation of HRR in ovarian cancer cell lines
Cells were incubated for 24 h in 20% O2 (N) and 0.1% O2 (H, hypoxic conditions). Lysates were Western blotted for PARP, RAD51, γH2AX, and vinculin as indicated. Methods detailed in Additional File 2.

No consistent changes of PARP-cleavage and gH2AX and in the expression of RAD51 protein were observed across a broad panel of cell lines (N=14), suggesting that hypoxia was not capable to weaken DNA repair.

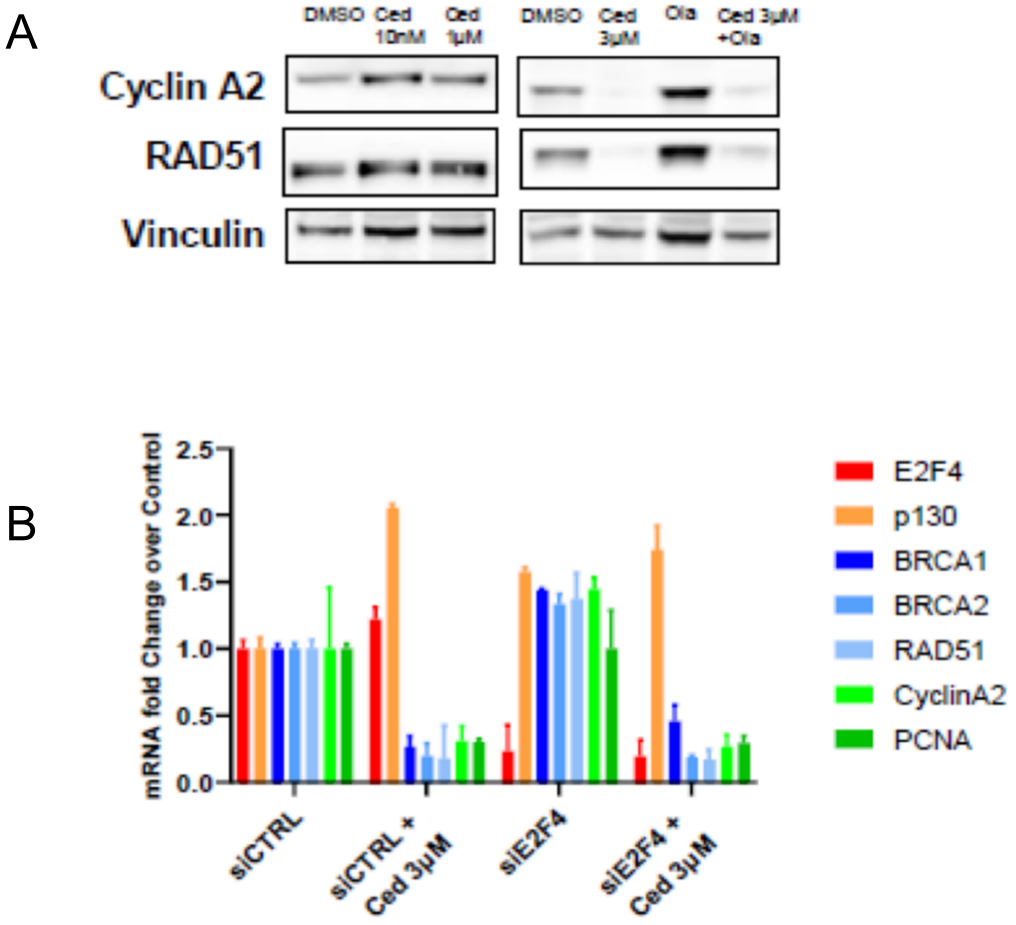

Supplementary Figure S9. Cediranib-mediated down regulation of RAD51 is not related to PDGFRB pathway
A Western blots show RAD51 and CyclinA2 in SKOV3 cells after treatment with cediranib (10 nM, 1 µM, 3 µM) or with the combination olaparib (3 µM) plus cediranib (3 µM).
B Expression of genes involved in HRR (*BRCA1*, *BRCA2*, *RAD51*) and cell cycle (CyclinA2, *PCNA*) after cediranib dosing (3 µM) by SKOV3 cells treated with siRNA to silence *E2F4* expression (siE2F4) or a scramble siRNA (siCTRL). Methods detailed in Additional File 2.

Cediranib 10 nM or 1 uM did not perturb the expression of RAD51 or CyclinA2, however RAD51 protein was reduced following 48 h of treatment with 3 uM cediranib. Silencing of E2F4 did not prevent the cediranib-mediated down regulation of RAD51 and of BRCA1 and BRCA2.

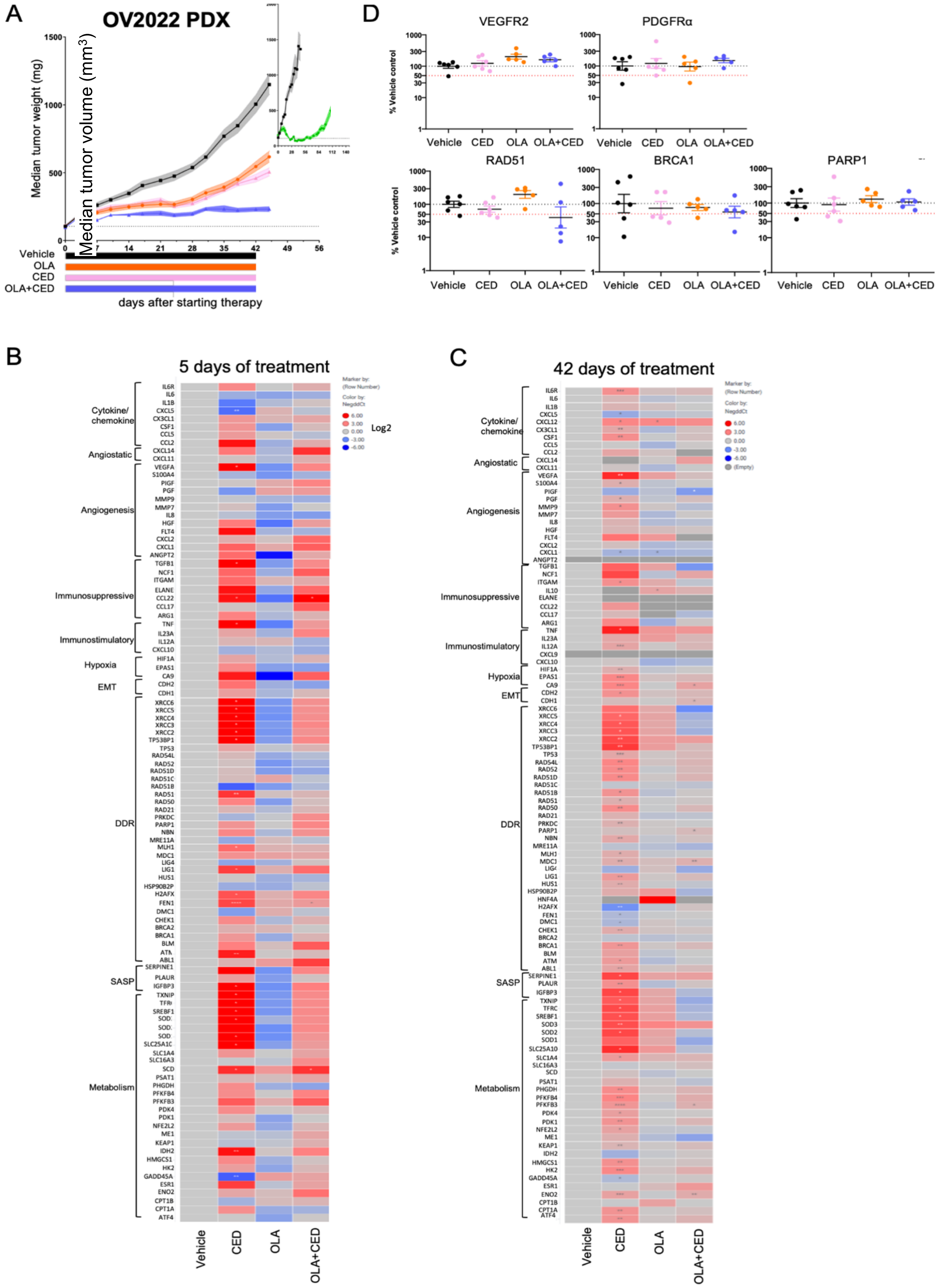

Supplementary Figure S10. Combination of olaparib and cediranib shows greater efficacy than either monotherapy in OV2022 tumours: no therapy-induced changes in HRR genes could be detected.

(See Legend on next page)

(*See Figure on previous page*)

Supplementary Figure S10. Combination of olaparib and cediranib shows greater efficacy than either monotherapy in OV2022 tumours: no therapy-induced changes in HRR genes could be detected.
A Mice bearing OV2022subcutaneous tumours were randomized to treatment at an average tumour volume of 104 mm^3 (SD 6.25), and treated with olaparib (100 mg/kg), cediranib (3 mg/kg) or with the combination for 6 weeks. Graphs are median tumour volume (mm3) ± median absolute deviation (MAD, shaded area). Coloured bars indicate the study dosing period. Response to carboplatin is reported in the insert.
B, **C** Heat map plot showing log2 expression of mRNA (4-5 mice per group; normalized to vehicle) after 5 days (**B**) or 6 weeks (**C**) of treatment. Fluidigm high-throughput gene expression analysis, methods detailed in Additional File 2. The tested genes are associated with metabolism, DNA damage repair (DDR), inflammation, angiogenesis, growth factor receptors, and cell phenotype such as epithelial-mesenchymal transition (ETM) and senescence-associated secretory phenotype (SASP), and are listed in Supplementary Table S1. Statistical analysis by t-test on the ddCt values. *P<0.05, **P<0.01.
D Densitometric analysis of Western blot detecting VEGFR2, PDGFR, RAD51 and BRCA1 protein in OV2022 tumours after 5 days of treatment. Geomean with SD is represented for each group where each dot represents a single tumour/mouse. All values were normalized to loading control.

Similar patterns of gene expression changes were detected following short and long term treatment. No reduction in expression of HRR genes (e.g. *BRCA* or *RAD51*) was detected, rather a modest increase was seen (B and C) which did not result in any significant difference of protein levels (D).

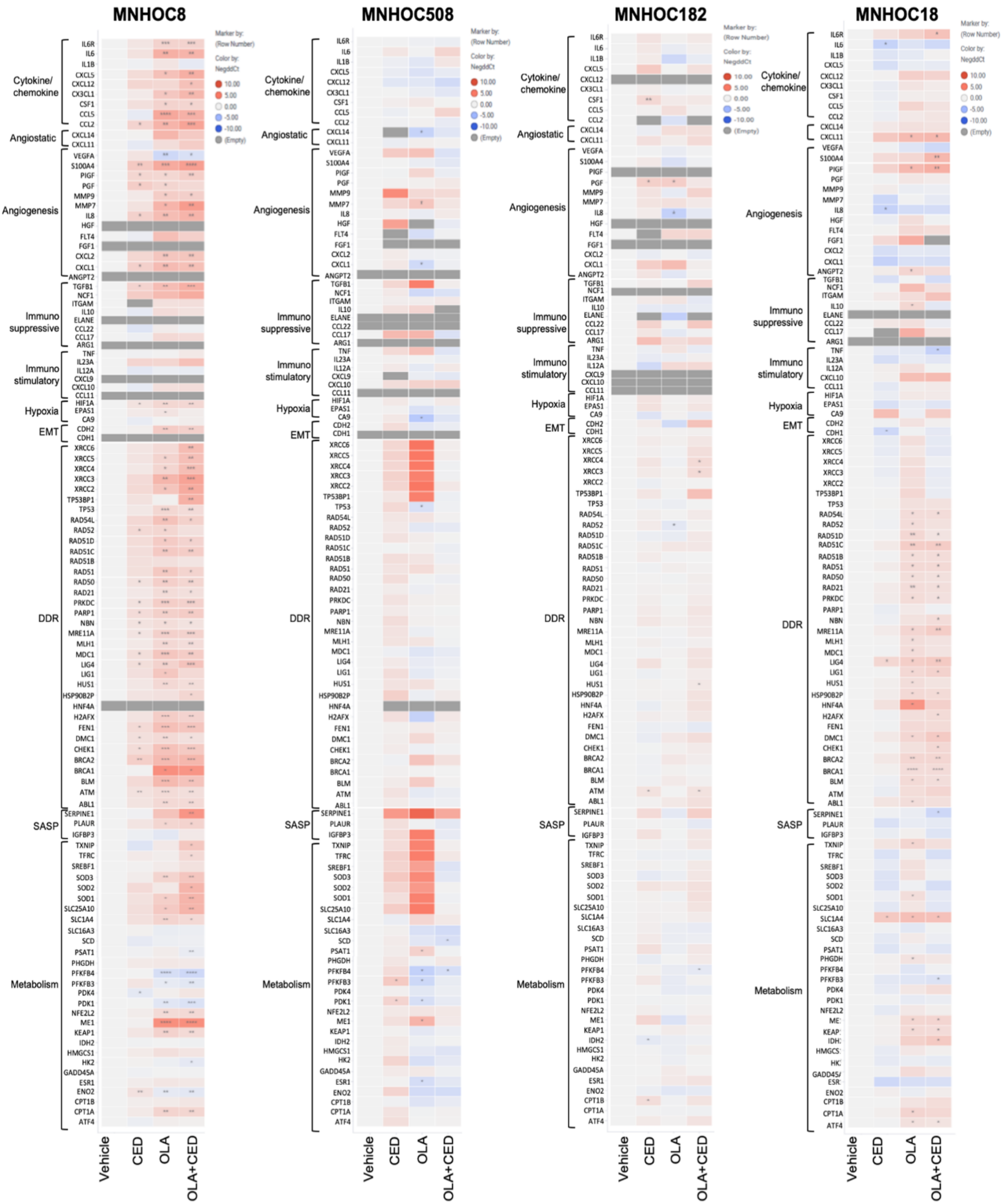

Supplementary Figure S11. No common changes in gene expression by cediranib treatment were identified in OC-PDXs that benefit from the combination therapy.

Heat map of mRNA expression assessed after 4 weeks of treatment. Three mice were analysed per group and each value represents median log2 gene expression normalized to vehicle group. Fluidigm high-throughput gene expression analysis, methods detailed in Additional File 2. The tested genes are as in Supplementary Fig. S10 and listed in Supplementary Table S1. Statistical analysis by t-test on the ddCt values. *P<0.05, **P<0.01, ***P<0.001.

Despite being significant in some cases, the changes in gene expression were not common across the OC-PDXs, downregulation of HRR genes could not be detected.

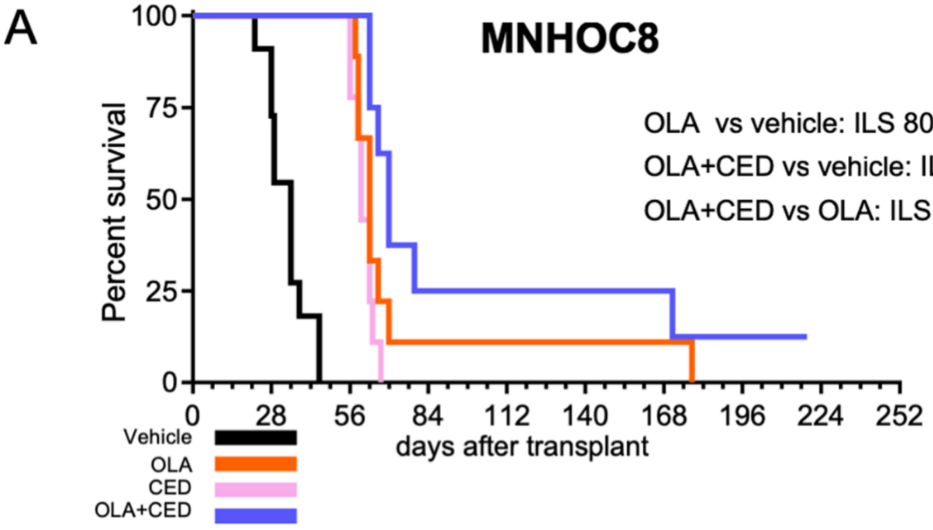

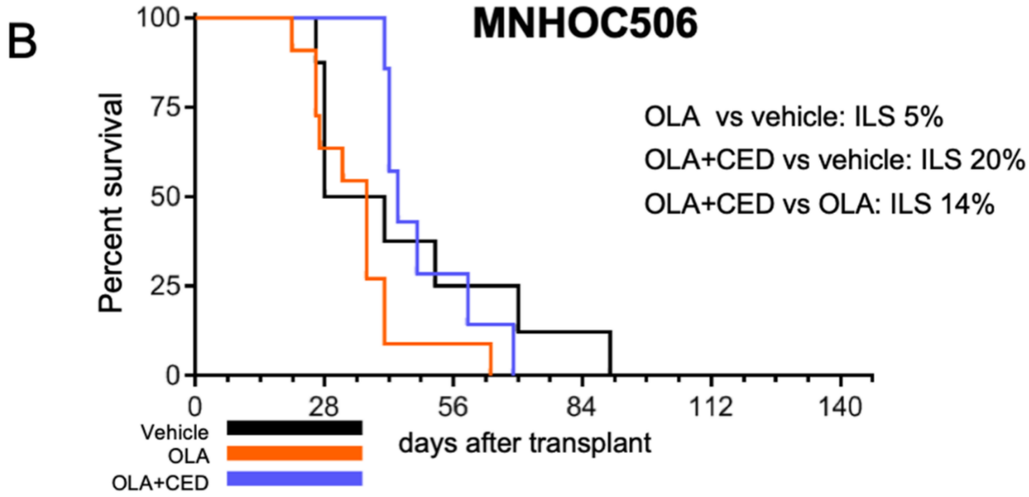

Supplementary Figure S12. Survival advantage is lost upon treatment interruption
A MNHOC8 and **B** MNHOC506 were transplanted intraperitoneally, mice were randomized and treated as described in Figure 2, with the difference that the treatment lasted only 4 weeks. Coloured bars indicate the study dosing period**.**

Supplementary Table S1. List of genes analysed by Fluidigm high-throughput gene expression analysis
DDR: DNA damage repair,
EMT: epithelial-mesenchymal transition,
SASP: senescence-associated secretory phenotype.

Gene	Gene Category	Species	Assay ID
18S	Housekeeping Gene	Human	Hs99999901_s1
ABL1	DDR	Human	Hs01104725_m1
ANGPT2	Angiogenesis	Human	Hs01048042_m1
ARG1	Immunosuppressive	Human	Hs00163660_m1
ATF4	Metabolism	Human	Hs00909569_g1
ATM	DDR	Human	Hs01112307_m1
BLM	DDR	Human	Hs01119886_g1
BRCA1	DDR	Human	Hs01556193_m1
BRCA2	DDR	Human	Hs01037421_m1
CA9	Hypoxia	Human	Hs00154208_m1
CCL11	Immunostimulatory	Human	Hs00237013_m1
CCL17	Immunosuppressive	Human	Hs00171074_m1
CCL2	cytokine/chemokine	Human	Hs00234140_m1
CCL22	Immunosuppressive	Human	Hs01574247_m1
CCL5	cytokine/chemokine	Human	Hs00982282_m1
CCNA2	Cell cycle	Human	Hs00996788_m1
CCND1	Cell cycle	Human	Hs00765553_m1
CDH1	EMT	Human	Hs01023894_m1
CDH2	EMT	Human	Hs00983056_m1
CHEK1	DDR	Human	Hs00967506_m1
CPT1A	Metabolism	Human	Hs00912671_m1
CPT1B	Metabolism	Human	Hs03046298_s1
CSF1	cytokine/chemokine	Human	Hs01094429_m1
CX3CL1	cytokine/chemokine	Human	Hs00171086_m1
CXCL1	Angiogenesis	Human	Hs00236937_m1
CXCL10	Immunostimulatory	Human	Hs00171042_m1
CXCL11	Angiostatic Genes	Human	Hs04187682_g1
CXCL12	cytokine/chemokine	Human	Hs00171022_m1
CXCL14	Angiostatic Genes	Human	Hs00171135_m1
CXCL2	Angiogenesis	Human	Hs00601975_m1
CXCL5	cytokine/chemokine	Human	Hs00171085_m1
CXCL9	Immunostimulatory	Human	Hs00171065_m1
DMC1	DDR	Human	Hs01095986_m1
E2F1	E2F	Human	Hs00153451_m1
E2F2	E2F	Human	Hs00231667_m1
ELANE	Immunosuppressive	Human	Hs00975994_g1
ENO2	Metabolism	Human	Hs00157360_m1
EPAS1	Hypoxia	Human	Hs01026138_m1

Cont on next page

ESR1	Metabolism	Human	Hs00174860_m1
FEN1	DDR	Human	Hs00748727_s1
FGF1	Angiogenesis	Human	Hs00361126_m1
FLT4	Angiogenesis	Human	Hs01047679_m1
FLT1	Angiogenesis	Human	Hs01052936_m1
GADD45A	Metabolism	Human	Hs00169255_m1
H2AFX	DDR	Human	Hs00266783_s1
HGF	Angiogenesis	Human	Hs00300159_m1
HIF1A	Hypoxia	Human	Hs00936368_m1
HK2	Metabolism	Human	Hs01034055_g1
HMGCS1	Metabolism	Human	Hs00940429_m1
HNF4A	DDR	Human	Hs04260079_mH
HPRT1	Housekeeping Gene	Human	Hs02800695_m1
HSP90B2P	DDR	Human	Hs00951544_s1
HUS1	DDR	Human	Hs00189595_m1
IDH2	Metabolism	Human	Hs00954278_m1
IGFBP3	SASP	Human	Hs00365742_g1
IL10	Immunosuppressive	Human	Hs00961622_m1
IL12A	Immunostimulatory	Human	Hs01073447_m1
IL1B	cytokine/chemokine	Human	Hs99999029_m1
IL23A	Immunostimulatory	Human	Hs00372324_m1
IL6	cytokine/chemokine	Human	Hs00985639_m1
IL6R	cytokine/chemokine	Human	Hs00794121_m1
IL8	Angiogenesis	Human	Hs00174103_m1
IPO8	Housekeeping Gene	Human	Hs00183533_m1
ITGAM	Immunosuppressive	Human	Hs00355885_m1
KEAP1	Metabolism	Human	Hs00202227_m1
KDR	Angiogenesis	Human	Hs00176676_m1
LIG1	DDR	Human	Hs01553527_m1
LIG4	DDR	Human	Hs01866071_u1
MDC1	DDR	Human	Hs00206182_m1
ME1	Metabolism	Human	Hs00159110_m1
MLH1	DDR	Human	Hs00179866_m1
MMP7	Angiogenesis	Human	Hs00159163_m1
MMP9	Angiogenesis	Human	Hs00957555_m1
MRE11A	DDR	Human	Hs00967443_m1
NBN	DDR	Human	Hs00159537_m1
NCF1	Immunosuppressive	Human	Hs00165362_m1
NFE2L2	Metabolism	Human	Hs00975961_g1
PARP1	DDR	Human	Hs00242302_m1
PDGFRA	Angiogenesis	Human	Hs00998018_m1
PDGFRB	Angiogenesis	Human	Hs00182163_m1
PDK1	Metabolism	Human	Hs01561850_m1
PDK4	Metabolism	Human	Hs01037712_m1
PFKFB3	Metabolism	Human	Hs00998700_m1

Cont on next page

PFKFB4	Metabolism	Human	Hs00190096_m1
PGF	Angiogenesis	Human	Hs01119259_m1
PHGDH	Metabolism	Human	Hs00198333_m1
PIGF	Angiogenesis	Human	Hs00903831_g1
PLAUR	SASP	Human	Hs00182181_m1
PRKDC	DDR	Human	Hs04195439_s1
PSAT1	Metabolism	Human	Hs00795278_mH
RAD21	DDR	Human	Hs00366721_mH
RAD50	DDR	Human	Hs00990023_m1
RAD51	DDR	Human	Hs00153418_m1
RAD51B	DDR	Human	Hs01568763_m1
RAD51C	DDR	Human	Hs00427442_m1
RAD51D	DDR	Human	Hs00979545_g1
RAD52	DDR	Human	Hs00172536_m1
RAD54L	DDR	Human	Hs00269177_m1
S100A4	Angiogenesis	Human	Hs01569256_m1
SCD	Metabolism	Human	Hs01682761_m1
SERPINE1	SASP	Human	Hs01126604_m1
SLC16A3	Metabolism	Human	Hs00358829_m1
SLC1A4	Metabolism	Human	Hs00983079_m1
SLC25A10	Metabolism	Human	Hs00201730_m1
SOD1	Metabolism	Human	Hs00533490_m1
SOD2	Metabolism	Human	Hs04260076_g1
SOD3	Metabolism	Human	Hs00162090_m1
SREBF1	Metabolism	Human	Hs02561944_s1
TEK	Angiogenesis	Human	Hs00945146_m1
TFRC	Metabolism	Human	Hs00951083_m1
TGFB1	Immunosuppressive	Human	Hs00171257_m1
TNF	Immunostimulatory	Human	Hs01113624_g1
TP53	DDR	Human	Hs01034249_m1
TP53BP1	DDR	Human	Hs00996818_m1
TXNIP	Metabolism	Human	Hs01006900_g1
VEGFA	Angiogenesis	Human	Hs00900055_m1
XRCC2	DDR	Human	Hs03044154_m1
XRCC3	DDR	Human	Hs00193725_m1
XRCC4	DDR	Human	Hs00243327_m1
XRCC5	DDR	Human	Hs00897854_m1
XRCC6	DDR	Human	Hs01922652_g1
