# Supplementary material for: VEGF pathway inhibition potentiates PARP inhibitor efficacy in ovarian cancer independent of BRCA status
Source: J Hematol Oncol. 2021 Nov 6;14:186. doi: 10.1186/s13045-021-01196-x (PMC8572452; doi:10.1186/s13045-021-01196-x)
Supplement: Supplementary file 2 — Additional file 2. Methods. [file 13045_2021_1196_MOESM2_ESM.pdf]

ADDITIONAL FILE 2

VEGF pathway inhibition potentiates PARP inhibitor efficacy in ovarian cancer independent of BRCA status.

Francesca Bizzaro, Ilaria Fuso Nerini, Molly A. Taylor, Alessia Anastasia, Massimo Russo, Giovanna Damia, Federica Guffanti, Francesca Guana, Paola Ostano, Lucia Minoli, Maureen M. Hattersley, Stephanie Arnold, Antonio Ramos-Montoya, Stuart C. Williamson, Alessandro Galbiati, Jelena Urosevic, Elisabetta Leo, Ugo Cavallaro, Carmen Ghilardi, Simon T. Barry, Maria Rosa Bani, Raffaella Giavazzi

METHODS

Xenograft tumor models

Female NCr-nu/nu mice, six to eight weeks old, were obtained from Envigo Laboratories (Udine, Italy), housed under specific pathogen-free conditions in the Institute's Animal Care Facilities and handled using aseptic procedures. Ectopic and orthotopic tumor models were selected from a previously established panel of OC-PDXs obtained as previously described [1,2]. Briefly, for ectopic models, tumor fragments (2-4 mm^3) were recovered from frozen stocks within 5-6 mouse passages after establishment from patients and engrafted subcutaneously in the flank of animals. For orthotopic OC-PDXs, tumor cell suspensions (8-10×10^6 cells) were injected intraperitoneally (i.p.) in nude mice. The description of OC-PDX is reported in [1,2] and completed here (Fig. S1 Additional File 1). OV2022 was originally sourced from Jackson Laboratories. Studies were performed according to UK Home Office and IACUC guidelines.

Drug preparation and administration

Olaparib (OLA, AZD2281, AstraZeneca, Alderley Park, Macclesfield, UK) was dissolved in 10% v/v DMSO in 10% w/v Kleptose (HP-β-CD) in purified deionized water and administered by oral gavage (p.o.) daily at 100 mg/kg (10 ml/kg) Cediranib (CED, AZD2171, AstraZeneca, Alderley Park, Macclesfield, UK) was suspended in 0.5% w/v HPMC (hydroxyl propyl methyl cellulose) 0.1% Tween80 (Sigma-Aldrich, Milan, Italy) in deionized water, vortexed and stirred overnight before dosing. Cediranib was given p.o. daily at 3 mg/kg (10 ml/kg). Olaparib and cediranib, alone or in combination, were given every day for 5 days on (Q1x5) and 2 days off until tumor progression, unless otherwise stated. In very responsive OC-PDXs treatments were stopped at regression and tumor re-growth monitored. Cisplatin (cis-diaminedichloroplatinum, DDP, Sigma-Aldrich, Milan, Italy) was dissolved in 0.9% NaCl and injected intravenously (i.v.) at the dose of 4

mg/kg (10 ml/kg). DDP was given once a week for 3 or 4 cycles. Vehicles were given with the same schedules and volumes as active compounds.

Antitumor activity

Tumor growth in subcutaneous models was measured with a Vernier caliper, and tumor volume was calculated as (length × width^2) / 2 = [mm^3]. OC-PDXs were randomized to treatment at approximately 280-330 mm^3 of tumor volume; OV2022 tumors were randomised at ~100mm^3. Mice were euthanized when tumor reached approximately 1500 mm^3 (and never beyond if it exceeded 15% of body weight). Tumor-free mice, as assessed by visual inspection approximately 200 days after the last dose, were considered cured. Differences in subcutaneous tumour volume were analysed by ANOVA and Tukey's post-test (or t test when only two groups were to be compared) all the days of measuring.

For orthotopic models, abdominal distension and palpable tumor masses in the peritoneal cavity were indicative of tumor growth [3]. Mice were randomized to treatment groups at an advanced stage (i.e. 25-30% of the expected median survival time), regularly monitored and euthanized as soon as they presented signs of discomfort (being considered the limit of survival) [3]. Survival time (ST) was recorded and Kaplan Meier curves generated. Increment of lifespan (ILS%) was calculated as [(median survival day of treated group − median survival day of control group) / median survival day of control group] x 100. A detailed autopsy was done to record the tumor burden in the peritoneal cavity. Pictures of the peritoneal cavity or organs were taken with a macro-digital imaging system (MacroPATH; Milestone S.r.l.). Tumor dissemination in representative organs of the peritoneal cavity (liver, diaphragm, omentum, pancreas, uterus/ovary, nodes) was ranked using an arbitrary score, previously described [3]: 0 = not infiltrated; 1 = small masses; 2 = evident masses; 3 = nearly completely invaded and 4 = completely invaded. Tumor burden was then calculated by summing the scores for each animal. Complete response, confirmed macroscopically at autopsy, was the absence of tumor in animals still alive on day 200 after transplant. Kaplan Meier survival curves were analyzed by Wilcoxon rank-sum test/log-rank test. Differences in abdominal tumor burden (ascites and dissemination scores) were analyzed by one way ANOVA and Tukey's post-hoc test.

Mutational analyses

Genomic DNA was obtained from tumor fragments or malignant ascites using a Maxwell® 16 Tissue DNA purification kit and the Maxwell® 16 Instrument for automated purification.

Whole Exome DNAseq

Two hundred nanograms of purified genomic DNA were used to construct a next generation sequencing (NGS) library. Libraries were generated using the Kapa Biosystems HyperPrep kit (following the manufacturer's protocol). All libraries were visualized on the Agilent TapeStation and the concentration was determined using the Kapa Biosystems NGS Library Quantification qPCR kit. Whole genome libraries were pooled and used for exome hybridization capture with xGen®Exome Research Panel v1.0 (Integrated DNA Technologies). Libraries were sequenced on the Illumina HiSeq 4000 platform (2 x 150) using TruSeq SBS (sequencing by synthesis) reagents (Illumina). The NGS data was aligned and analyzed within the BCBio framework (https://bcbio-nextgen.readthedocs.org/en/latest/) using an AstraZeneca-developed variant calling algorithm (https://github.com/AstraZeneca-NGS/VarDict) [4]. For this study, 31 genes involved in homologous recombination repair (HRR) were analyzed (30 genes as reported in [5] plus FANCB).

Sanger sequencing

DNA (or mRNA/cDNA) were amplified with a GoTaq® PCR Core System (Promega), using a thermocycler (2720 Thermal Cycler, Applied Biosystem) and *ad hoc* primers designed to detect specific mutations and amplify human but not murine variants (lack of amplification of purified mouse DNA (or mRNA/cDNA) was the benchwork technical control). PCR products were purified with Illustra™ GFX PCR and Gel Band Purification kit (GE Healthcare). and sequenced by Microsynth SeqLab (Switzerland). Electropherograms were analyzed, and mutations confirmed using Sequencer 5.1 software and FinchTV software.

Gene expression

Total RNA was extracted from tumor fragments or malignant ascites using QIAzol Lysis Reagent and Tissuelyser LT Adapter (Qiagen) and purified through RNeasy® mini-Kit with the QIAcube® robotic workstation (Qiagen). The concentration and purity of nucleic acid samples were determined by spectrophotometric analysis (Nanodrop® spectrophotometer).

RNA-seq

RNA integrity was further checked using Agilent 6000 Nano Assay (Agilent Bioanalyzer®), considering only RIN numbers of 6 or more acceptable. RNA was quality re-validated using the Agilent TapeStation and Quant-iT RNA Assay Kit (Thermo Fisher) before submitting the totRNA for eukaryotic RNA-seq library prep and paired-end sequencing (50M reads/sample) on the Illumina HiSeq Platform at Novogene. Quality control of raw reads was done with the FastQC 0.11.9 tool. Adapters and low-quality reads were trimmed using Cutadapt 2.9 and the reads were then mapped on the Human Reference Genome GRCh38.99 using Star 2.6. The mapped reads were counted with FeatureCounts 2.0. Finally, the DeSeq2 package, available within Bioconductor, was

used to normalize counts, estimate biological variance and determine differential expression. Log2-fold changes and adjusted p-values were generated for each class comparison. A heatmap representation of RNA-seq data was plotted using MeV version 4.9.0.

Fluidigm high-throughput analysis

cDNA was synthesized using cDNA Preparation with Fluidigm® Reverse Transcription Master Mix following manufacturer's instructions. Gene expression pre-amplification was performed using Fluidigm Preamp Master Mix as per manufacturer's instructions. 96x96 Fluidigm chips were primed using the Juno™ instrument and upon priming samples and primers were loaded and the reaction was run using the BioMark™ HD System (Fluidigm). The list of TaqMan™ (Applied Biosystems) probes used is provided in Supplementary Table 1. Raw data was analysed using Fluidigm Real Time PCR Analysis Software. Data was uploaded onto a R Shiny Tool, using a Tool Template (AstraZeneca) QC'ed and exported. dCt was calculated in R Shiny Tool by taking Ct - Ct housekeeper with the least variability across the experiment (or average of multiple housekeepers) for each gene. An average dCt for all vehicle controls was calculated (dCt - dCt(avg. vehicle)) and used to calculate negative ddCt. 2^negativeddCt was used to calculate Fold Change. P values were calculated by performing a Students t-test on the negative ddCt values. Data was plotted using Spotfire software.

Western blotting

Tumor samples were lysed in lysis buffer (20 mM Tris pH7.5, 137 mM NaCl, 10% Glycerol, 50 mM NaF, 1 mM Na_3VO_4, 1% SDS, 1% NP40 supplemented with protease inhibitors (Roche, Basel, Switzerland) and phosphatase inhibitors (Sigma-Aldrich)) and homogenised using a FastPrep-24™ machine (MP Biomedicals) respectively. Equal amounts of whole-cell protein extracts (40 μg) were separated on 3-8% Tris-Acetate NuPAGE gels and analysed by standard Western blotting. The following antibodies were used: rabbit polyclonal antibodies against BRCA1 (Merck Millipore no 07-434; dilution 1:500), rabbit polyclonal against PARP1 (Cell Signaling 9542; dilution 1:1000), mouse monoclonal antibody against VEGFR2 (ThermoFisher A5-15556; dilution 1:500), rabbit monoclonal antibody against RAD51 (Cell Signaling 8875; dilution 1:500), goat polyclonal against PDGFRα (R&D Systems AF307; dilution 1:500), mouse monoclonal against vinculin (Sigma V4505, dilution 1:5000).

Immunohistochemistry

For immunohistochemical analyses, solid tumors fragments were collected after 28 days of therapy, 6 h after the last dose. Tumors and organs were fixed in 10% neutral buffered formalin and paraffin-

embedded, and 4-μm serial sections were prepared.

Deparaffinisation, rehydration and antigen retrieval were done in a single step, immersing sections in Dewax and HIER Buffer H (Thermo Scientific) for 40 min at 94°C, followed by incubation with 3% H_2O_2 for 15 min to block endogenous peroxidase activity and treatment with 10% normal rabbit serum for 30 min to reduce the nonspecific background staining, with another incubation for 1 h with anti-CD31 antibody (monoclonal, rat, SZ31 Dianova DIA310). Biotinylated rabbit anti-rat secondary antibody (Vector Laboratories) was then added, incubated for 30 min, then labeled by the avidin-biotin-peroxidase (ABC) procedure with a commercial immunoperoxidase kit (Vectastain Standard Elite, Vector Laboratories). The immunoreaction was revealed with 3,3'-diaminobenzidine substrate (DAB, Vector Laboratories) for 5 min and sections were counterstained with Mayer's hematoxylin. Microvessel density (MVD) was established by computing the number of CD31-positive vessels in three 200x microscopic fields, randomly selected throughout the neoplastic tissue, using ImageJ software [6]. MVD differences were analyzed by one way ANOVA and Tukey's post-hoc test.

Cell line culture

Ovarian cancer cell lines were originally obtained from ATCC. Cell line identification was validated using the CellCheck assay (IDEXX Bioanalytics, Westbrook, ME, USA). All cell lines were validated free of mycoplasma contamination using the MycoSEQ assay (Thermo Fisher Scientific, Waltham, MA, USA) or STAT-Myco assay (IDEXX Bioanalytics). Cell lines were grown in RPMI-1640 growth media (Corning 17-105-CV) supplemented with 10% fetal bovine serum (FBS) and 2 mM glutamine. SKOV3 cell lines were grown in McCoy's 5a Medium (Gibco, 16600082) with 10% fetal bovine serum and 1% L-glutamine. For in vitro studies, cediranib and olaparib (AstraZeneca, Cambridge, UK) were solubilized in DMSO.

Effect of hypoxia in cell lines

To determine the effect of hypoxia on the expression of HRR proteins, the panel of ovarian cell lines (N=14) were incubated in standard media in the presence of either 20% oxygen or 0.1% oxygen for 24 h in a Baker Ruskinn (Maine, USA) InvivoO_2 hypoxic chamber for 24 h. Cells were then lysed in 20 mM Tris (pH 7.5), 137 mM NaCl, 10% Glycerol, 50 mM NaF, 1 mM Na_3VO4, 1% SDS, 1% NP40 substitute, protease inhibitors (Roche) and phosphatase inhibitor cocktails 2 and 3 (Sigma) on ice (4^oC). Lysates were sonicated, clarified by centrifugation. Equal amounts of protein were then Western blotted for PARP (CST cat 9542L), RAD51 (CST cat D4B10), γH2AX (CST cat 9718), Vinculin (Abcam cat ab18058), and detected using the appropriate goat anti-mouse or -rabbit

HRP conjugated secondary antibodies and chemiluminescence reagent. Signal was imaged on a Gbox (Syngene, Maryland USA).

Cell proliferation assay to assess cediranib-mediated sensitization of cells to olaparib

Cells were plated in 96-well plates, allowed to attach overnight and were then dosed using the HP D300e Digital Dispenser (HP Life Science Dispensing) at the indicated concentrations of cediranib and olaparib. Cells were allowed to proliferate for 7 days at 37°C in standard culture media in the presence or absence of the relevant compound. To determine proliferation a live cell count post-treatment was determined using a Sytox Green endpoint as previously described [7], and a CellTiter-Glo as per manufacturer's instructions (Promega, Madison, WI, USA; G7570). Data were normalized to the vehicle treated samples and dose-response curves were plotted in GraphPad prism V8.3 using the nonlinear regression model. For each biological repeat, cell proliferation values were averaged from two technical replicates. In the represented plots, error bars are mean ± S.E.M for three biological replicates.

Assessment of regulation of RAD51 expression in SKOV3

To analyze changes in protein expression following treatment with cediranib, SKOV3 cells were lysed in RIPA buffer (Sigma-Aldrich) supplemented with protease inhibitors (Roche, Basel, Switzerland) and phosphatase inhibitors (Sigma-Aldrich). Equal amounts of whole cell lysates were separated on 4-12% Bis-Tris NuPAGE gels and analysed by standard immunoblotting. Antibodies used were RAD51 (70-001, 1:6000) from BioAcademia (Osaka, Japan), Cyclin A2 (ab38, 1:500) from Abcam (Cambridge, UK), Vinculin (V9131, 1:2000) from Merck Millipore (Burlington, MA, USA). Immunoblots are representative of experiments that were performed at least twice. To downregulate *E2F4* gene silencing with siRNA transfection was performed. Cells were seeded in 6-well plates and transfected with siE2F4 (Horizon Discovery Ltd, Cambridge, UK L-003262-00-0010) or non-targeting siCTRL (Horizon Discovery Ltd, Cambridge, UK, D-001810-0X), using the RNAiMax transfection reagents (ThermoFisher Scientific, 13778075), following manufacturer instructions, at a final concentration of 10nM. Cells were incubated with the siRNA, RNAiMAX, OPTIMEM (ThermoFisher Scientific, 31985070) and the appropriate cell culture medium for 48 h.

To analyze changes in gene expression, total RNA was isolated from cells using the RNeasy Kit (QIAGEN, 74104), according to the manufacturer's instructions to a final volume of 40 μL per well. Gene expression of genes were evaluated by qPCR using the ONE-step QuantiTect Probe RT-PCR kit (Qiagen, Cat No./ID: 204445). For each reaction 2 μL of RNA were used. RT-qPCR

reactions were performed on a Roche Lightcycler 480 II Sequence Detection System. The following Taqman probes were obtained by ThermoFisher Scientific: *E2F4* (Hs00608098_m1, cat no. 4331182), *RBL2* (p130, Hs00180562_m1, cat no. 4331182), *BRCA1* (Hs01556193_m1, cat no. 4331182), *BRCA2* (Hs00609073_m1, cat. No. 4331182), *RAD51* (Hs00947967_m1, cat. No. 4331182), *CCNA2* (CyclinA2, Hs00996788_m1, cat. No. 4331182), *PCNA* (Hs00427214_g1, cat. No. 4331182).

References

1. Ricci F, Bizzaro F, Cesca M, Guffanti F, Ganzinelli M, Decio A, et al. Patient-derived ovarian tumor xenografts recapitulate human clinicopathology and genetic alterations. Cancer Res. 2014;74:6980–90. https://doi.org/10.1158/0008-5472.CAN-14-0274

2. Guffanti F, Fratelli M, Ganzinelli M, Bolis M, Ricci F, Bizzaro F, et al. Platinum sensitivity and DNA repair in a recently established panel of patient-derived ovarian carcinoma xenografts. Oncotarget. 2018;9:24707–17. https://www.ncbi.nlm.nih.gov/pubmed/29872499

3. Oliva P, Decio A, Castiglioni V, Bassi A, Pesenti E, Cesca M, et al. Cisplatin plus paclitaxel and maintenance of bevacizumab on tumour progression, dissemination, and survival of ovarian carcinoma xenograft models. Br J Cancer. 2012;107:360–9. https://doi.org/10.1038/bjc.2012.261

4. Lai Z, Markovets A, Ahdesmaki M, Chapman B, Hofmann O, McEwen R, et al. VarDict: a novel and versatile variant caller for next-generation sequencing in cancer research. Nucleic Acids Res. 2016;44:e108. https://doi.org/10.1093/nar/gkw227

5. Coleman RL, Oza AM, Lorusso D, Aghajanian C, Oaknin A, Dean A, et al. Rucaparib maintenance treatment for recurrent ovarian carcinoma after response to platinum therapy (ARIEL3): a randomised, double-blind, placebo-controlled, phase 3 trial. Lancet. 2017;390:1949–61. https://doi.org/10.1016/S0140-6736(17)32440-6

6. Schneider CA, Rasband WS, Eliceiri KW. NIH Image to ImageJ: 25 years of Image Analysis. Nat Methods. 2012;9:671–5. https://doi.org/10.1038/nmeth.2089

7. Fok JHL, Ramos-Montoya A, Vazquez-Chantada M, Wijnhoven PWG, Follia V, James N, et al. AZD7648 is a potent and selective DNA-PK inhibitor that enhances radiation, chemotherapy and olaparib activity. Nat Commun. 2019;10:5065. https://doi.org/10.1038/s41467-019-12836-9
